# Developing public health risk messages about antibiotic resistance using metaphors: an international co-design and e-Delphi consensus study

**DOI:** 10.1038/s41598-026-40577-5

**Published:** 2026-02-18

**Authors:** Eva M. Krockow, Meghann Jones, Samkele Mkumbuzi, Marc Mendelson, Carolyn Tarrant, Robert Froud, Anastasia Koch, Stephen J. Flusberg, Emma Pitchforth

**Affiliations:** 1 School of Psychology and Vision Sciences, University of Leicester, University Road, Leicester, LE1 7RH UK; 2 Division of Infectious Diseases & HIV Medicine, Department of Medicine, Groote Schuur Hospital, University of Cape Town, Cape Town, South Africa; 3 School of Medical Sciences, University of Leicester, Leicester, UK; 4 National Institute for Health and Care Research (NIHR) Greater Manchester Patient Safety Research Collaboration (GM PSRC), Manchester, UK; 5 School of Health Sciences, Kristiania University of Applied Sciences, Oslo, Norway; 6 Warwick Medical School, University of Warwick, Coventry, UK; 7 Eh!woza, Khayelitsha, South Africa; 8 Molecular Mycobacteriology Research Unit, Institute of Infectious Disease and Molecular Medicine and Department of Pathology, University of Cape Town, Cape Town, South Africa; 9 Department of Cognitive Science, Vassar College, Poughkeepsie, NY USA; 10 Faculty of Health and Life Sciences, University of Exeter, Exeter, UK

**Keywords:** Antimicrobial resistance, Antibiotic resistance, Drug-resistant infections, Public health, Risk communication, Metaphor, Analogy, Health care, Medical research

## Abstract

Antimicrobial resistance (AMR) is a global health threat, yet public awareness of its causes and risks remains low, limiting behaviour change. Metaphors—linking abstract ideas to familiar concepts—are promising but underused tools in AMR communication. We conducted the first systematic mixed-methods study to develop novel metaphors addressing key misunderstandings and behaviours. Co-design workshops with the public and doctors in the UK (*n* = 29) and public in South Africa (*n* = 22) generated 89 initial metaphors. These were extended through 101 additional suggestions, and evaluated via a 3-round e-Delphi study with 37 AMR communication experts from 27 countries, using the UCLA/RAND appropriateness method. 38 metaphors were endorsed for global use. Invoking domains like nature, tools, and fire fighting, they offer relatable, culturally resonant alternatives to alarmist war and doomsday imagery. This study pioneers participatory methods in behavioural science and provides a blueprint for co-creating health messages, such as tailoring AMR metaphors to specific needs.

**Supplementary Information:**

The online version contains supplementary material available at 10.1038/s41598-026-40577-5.

## Introduction

Antimicrobial resistance (AMR)—the adaptive survival of microbes against medical treatments including antibiotics—is an ancient evolutionary mechanism that has enabled bacterial survival for billions of years—yet its growing impact today represents a pressing global health emergency. AMR develops when microbes undergo genetic changes and face conditions that favour the survival of those changes. These shifts—whether from mutations or gene sharing—allow some microbes to withstand drugs that once killed them, and those survivors multiply and spread. With few new antimicrobial drugs on the horizon, AMR has become a significant driver of morbidity and mortality. In 2019, antibiotic resistance alone was linked to an estimated 1.27 million deaths worldwide^1^, with forecasts predicting 39.1 million attributable deaths in the next 25 years^2^. Human behaviour is increasingly recognised as pivotal to safeguarding existing antibiotics for future generations, particularly in light of challenges in antibiotic development and modest advances in diagnostics^3^. Any use of antibiotics contributes to the problem of AMR and compromises the public good of antibiotic efficacy. This makes it a resource conservation challenge^4 ^or social dilemma akin to climate change^5^. The general public, as part of multiple and dynamic publics^6^, play a crucial role in this dilemma, given their profound influence on antibiotic use—whether through individual health behaviours, shared decision making with healthcare providers, or wider societal support for policy initiatives and investments that accelerate antibiotic development and innovation. Yet, shortcomings in AMR communications remain a persistent barrier to public awareness and knowledge uptake^7^, both of which are essential precursors to promoting positive behavioural change^8^.

Surveys consistently reveal widespread knowledge gaps and misunderstandings about AMR among lay populations. These include misbeliefs that antibiotics are effective against common illness symptoms (e.g., diarrhoea)^9 ^or viral infections^10^, that resistance occurs in the human body rather than in bacteria^11^, and that antibiotics are necessary, even in cases of minor infections that could resolve without treatment^12^. Additionally, evidence points to many behaviours that reflect a lack of understanding including the sharing of leftover antibiotics^13^, premature treatment termination without prior medical consultation^14^, self-medication, and general non-adherence to medical advice^15^. Finally, there remains a lack of awareness about the relationship between infection prevention (e.g., through vaccination, hand hygiene, and broader infection control) and AMR^15^.

Past communication efforts have been criticised for relying on abstract, scientific language, personally or culturally irrelevant messages, and ineffective framing^7,16–18^. In response, there has been growing momentum for clearer, more relatable approaches to risk messaging. Noteworthy initial examples include the Wellcome Trust’s ‘Reframing resistance’ report, which provides research-driven recommendations for strategic framing^7^, modular messaging guides—often accompanied by social media toolkits—developed by international organisations and charities like the UN Foundation^19 ^and the AMR Narrative^20^, and increasingly creative public health campaigns. For example, Japan used anime heroes for broader public appeal^21 ^and the UK recently launched a digital ‘Andi Biotic’ campaign, featuring a life-sized talking pill mascot^22^. While useful as a starting point, more work is needed to build a diverse, evidence-driven toolbox of engaging and actionable messages for successful global AMR communications.

Metaphor represents a promising, yet under-explored tool in the context of AMR communications. Metaphors can make unfamiliar, abstract concepts more comprehensible by describing them using more familiar and concrete terms^23^. This enables someone to use what they already know to reason about a novel domain that might otherwise be difficult to understand. For instance, comparing vaccines to *training sessions* for the body’s natural *defence troops*—antibodies and memory B and T cells—may help to clarify how vaccines work. This metaphor also highlights the importance of booster shots, which refresh immune system *preparedness* and provide updated *intelligence* on evolving threats^24^. Strategic metaphorical framing with a clear intent therefore has the potential to shift perspectives, change attitudes and motivate behaviour change^25^. On top of helping people conceptualise complex concepts, metaphors are emotionally engaging and memorable and may facilitate social communication^25,26^.

While extensively studied in messaging around cancer^27,28^, COVID-19^29,30^, and vaccine hesitancy^24,31^, insights into strategic metaphorical framing of AMR remain limited. Furthermore, with research pointing to the potential harm of metaphors—particularly in cross-cultural communication, when speakers share limited mutual understanding of the metaphorical source domain^32^—carefully considered, evidence-based use of metaphors is likely essential for communication success.

Content analyses of written AMR discourse have revealed a reliance on conventional imagery—such as war and doomsday scenarios—which often lack novelty and apt metaphorical mappings^33–35^. Frequent references to *invincible superbugs*, a *silent pandemic* or *tsunami* of AMR or an imminent return to the *pre-antibiotic era* or *medical dark ages* may at best communicate a sense of urgency, yet fail to equip audiences with actionable recommendations on how to address the threat^33^. At worst, they could undermine people’s sense of agency in solving the problem, leading them to disengage completely.

**Fig. 1:**
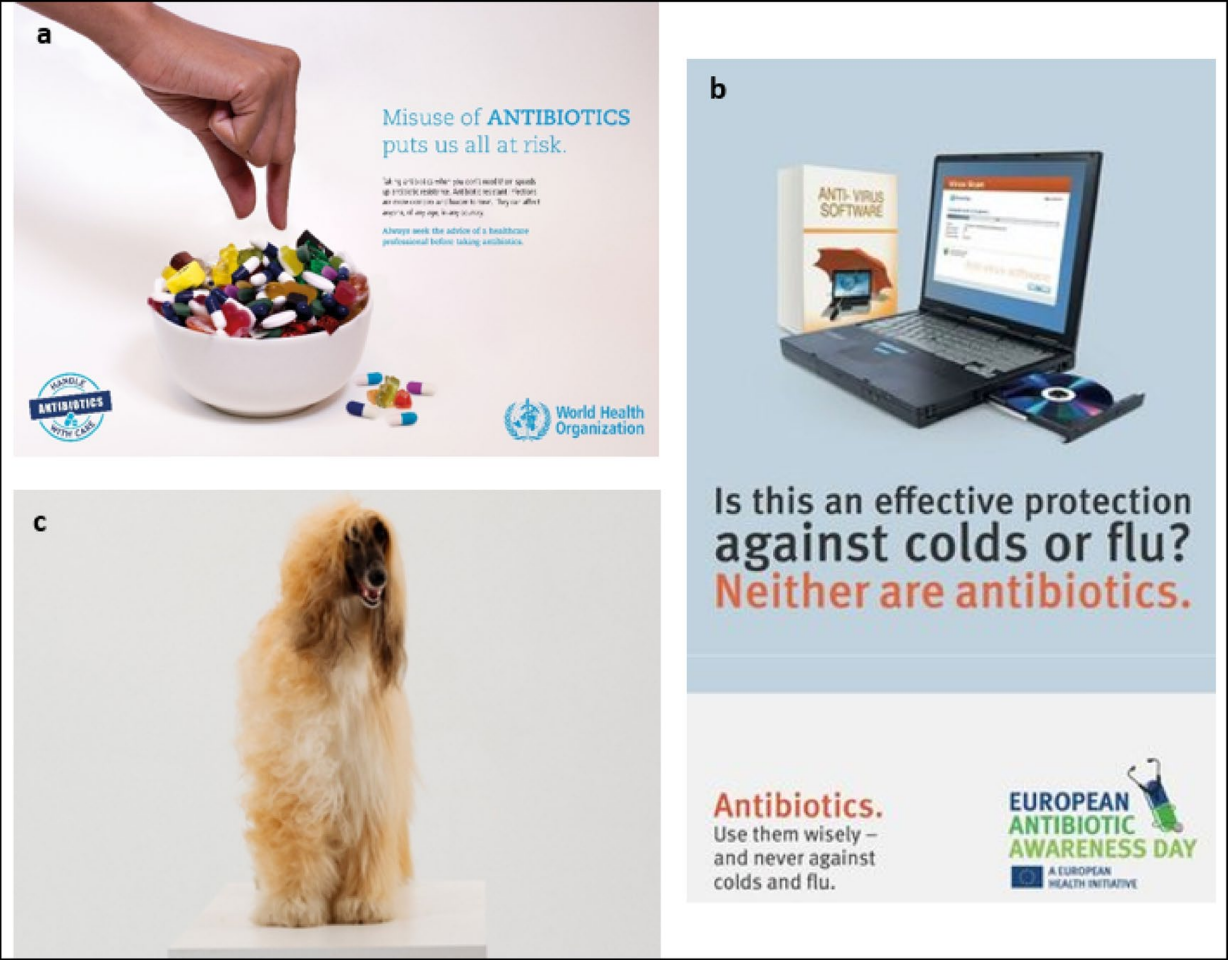
Examples of previous public health campaign materials about AMR incorporating visual metaphors. (**a**) Metaphorical comparison of antibiotic misuse with casual consumption of sweets as part of the ‘Handle antibiotics with care campaign’, image courtesy of WHO, used with permission, campaign available at: https://www.who.int/news-room/events/detail/2017/11/13/default-calendar/world-antibiotic-awareness-week-2017. (**b**) Metaphorical comparison of antibiotic use and virus software for treating viral infections as part of the 2017 European Antibiotic Awareness Day campaign, image adapted from ECDC, under CC BY 4.0, campaign available at: https://antibiotic.ecdc.europa.eu/en/poster-effective-protection-against-colds-or-flu-neither-are-antibiotics-antivirus. (**c**) Metaphorical comparison of an incomplete antibiotics course to a half-groomed dog as part of the ‘Don’t Leave It Halfway’ campaign from the first edition of the European Joint Action on Antimicrobial Resistance and Healthcare-Associated Infections (EU-JAMRAI) funded by the former Consumers, Health, Agriculture and Food Executive Agency (CHAFEA) of the European Commission under the Grant Agreement 761,296, used with permission, campaign available at: https://eu-jamrai.eu/jamrai1/dontleaveithalfway/.

Some public health campaigns have incorporated visual metaphors, often without explicit strategic intent (Fig. 1). For example, the 2017 WHO Antibiotic Awareness Week campaign included imagery depicting antibiotics mixed with sweets, serving as a metaphor for the indiscriminate and casual consumption of antibiotics^36^. A 2017 poster from the European Centre for Disease Prevention and Control (ECDC)^37 ^suggested that using antibiotics are as ineffective as antivirus computer software for treating human viral infections such as colds and flu. Another example is the 2018 ‘Don’t leave it halfway’ initiative^38 ^by the European Joint Action on Antimicrobial Resistance and Healthcare-Associated Infections (EU JAMRAI). This campaign featured short videos comparing incomplete antibiotic treatment courses to different humorous scenarios such as a half-groomed dog or a concert abruptly abandoned by the lead performer. While previous visuals captured attention, their effectiveness in conveying the seriousness of antibiotic misuse or offering clear behavioural solutions was unclear. For example, a dog with lopsided fur may appear comical, thereby downplaying the potentially serious consequences of non-adherence to treatment advice. Moreover, the long-standing recommendation to always finish a full course of antibiotics without question is now considered outdated. Current guidance emphasises the importance of taking antibiotics as directed and seeking medical advice before stopping treatment^39,40^. This shift reflects growing evidence that shorter antibiotic courses can be just as effective^41,42^, and acknowledges that many prescriptions may have been unnecessary to begin with—making reduced durations both safer and more appropriate.

To date, there has been no systematic attempt to develop novel metaphors that are both engaging and theoretically apt to communicate actionable messages about AMR and thereby motivate behaviour change.

Given the global nature of AMR, any attempt to overcome existing communication challenges through metaphors requires coordinated international efforts to establish shared principles that can be locally adapted. Additionally, there is a recognised need to involve patients, public, and key stakeholders in the design of AMR messages—extending beyond traditional scientific methods to embrace co-production and participatory research^43^. These stakeholders bring with them personal experiences and worldviews that offer alternative ways of thinking—disrupting the deeply entrenched war metaphors that have historically framed medical practice and approaches to illness^44^. We responded to the need of stakeholder involvement through a mixed-methods study that used a novel, cross-cultural participatory metaphor design procedure. While prior efforts have crowdsourced metaphor ideas to reframe COVID-19 communication^45^, this study is—to our knowledge—the first to engage public stakeholders, professionals, and international topic experts in a systematic process of novel metaphor generation. We conducted co-design workshops in the UK and South Africa and an international e-Delphi study to generate, evaluate, and refine AMR metaphors on a global scale. Our work addressed the following central research question: What types of metaphors do relevant stakeholders and expert AMR communicators judge as most appropriate for public-facing risk communication about antibiotic resistance?

## Results

### Co-design

UK co-design workshops, conducted separately with doctors and members of the public, comprised 29 participants across 7 workshops (14 doctors, 15 members of the public; 13 male, 16 female; mean age = 44.91 years, *SD* = 16.87). One larger co-design workshop in South African included 22 participants (7 male, 15 female; mean age = 40.64 years, *SD* = 11.24). For more detailed demographic data see Supplementary Note 1.

The initial metaphors generated varied in volume and thematic range. UK workshops with members of the public generated metaphors with a strong focus on dispelling the misconception that antibiotics treat viral infections. Participants readily identified everyday futile or harmful actions—such as eating soup with a fork or using aftershave on sunburnt skin—often incorporating humour (e.g., a woman using a man’s urinal). When trying to communicate mechanisms of AMR, war metaphors (e.g., fighting soldiers) were common. However, most participants struggled to convey that it is bacteria—not the human body—that develop resistance. Some found metaphor generation challenging altogether, with the required level of abstraction proving difficult. In several cases, metaphors drawn from healthcare contexts (e.g., using cough sweets to cure pneumonia) were excluded from the e-Delphi study due to insufficient conceptual distance between source and target domains. UK workshops with doctors showed similar variability. Their contributions often built on patient suggestions and expanded to include metaphors for inappropriate antibiotic use involving tools (e.g., wrong key in a lock, blunt knife), and metaphors explaining principles of AMR including video games (e.g., bacteria as opponents), and sports (e.g., opposing teams learning strategies). War metaphors remained prevalent.

A South African co-design workshop yielded more culturally-specific metaphors, often with themes rooted in local experiences and idioms. The invisibility of AMR was likened to the absence of a father figure. Prematurely discontinuing an antibiotic course without consulting a healthcare professional was perceived as a self-inflicted vulnerability—akin to living in a house without a roof, exposed and unprotected. Similarly, inappropriate antibiotic use was likened to self-sabotage, captured in the isiXhosa idiom “Ukuzibetha Ngenyheke elityeni” (hitting oneself with a rock on the lip). Some metaphors referenced culturally and politically sensitive topics, which were excluded from the e-Delphi study due to their potentially controversial nature. One metaphor—comparing inappropriate antibiotic use to filling a diesel car with petrol—emerged independently in both settings. A total of 89 metaphors were identified as suitable candidates for rating in the subsequent e-Delphi study (see Supplementary Note 2). These were organised into nine thematic sections that aligned with knowledge gaps identified in previous literature and incorporated specific contributions from co‑design participants—for instance, unprompted comments and metaphor suggestions emphasising the role of infection prevention in addressing antibiotic resistance.

### e-Delphi

**Fig. 2:**
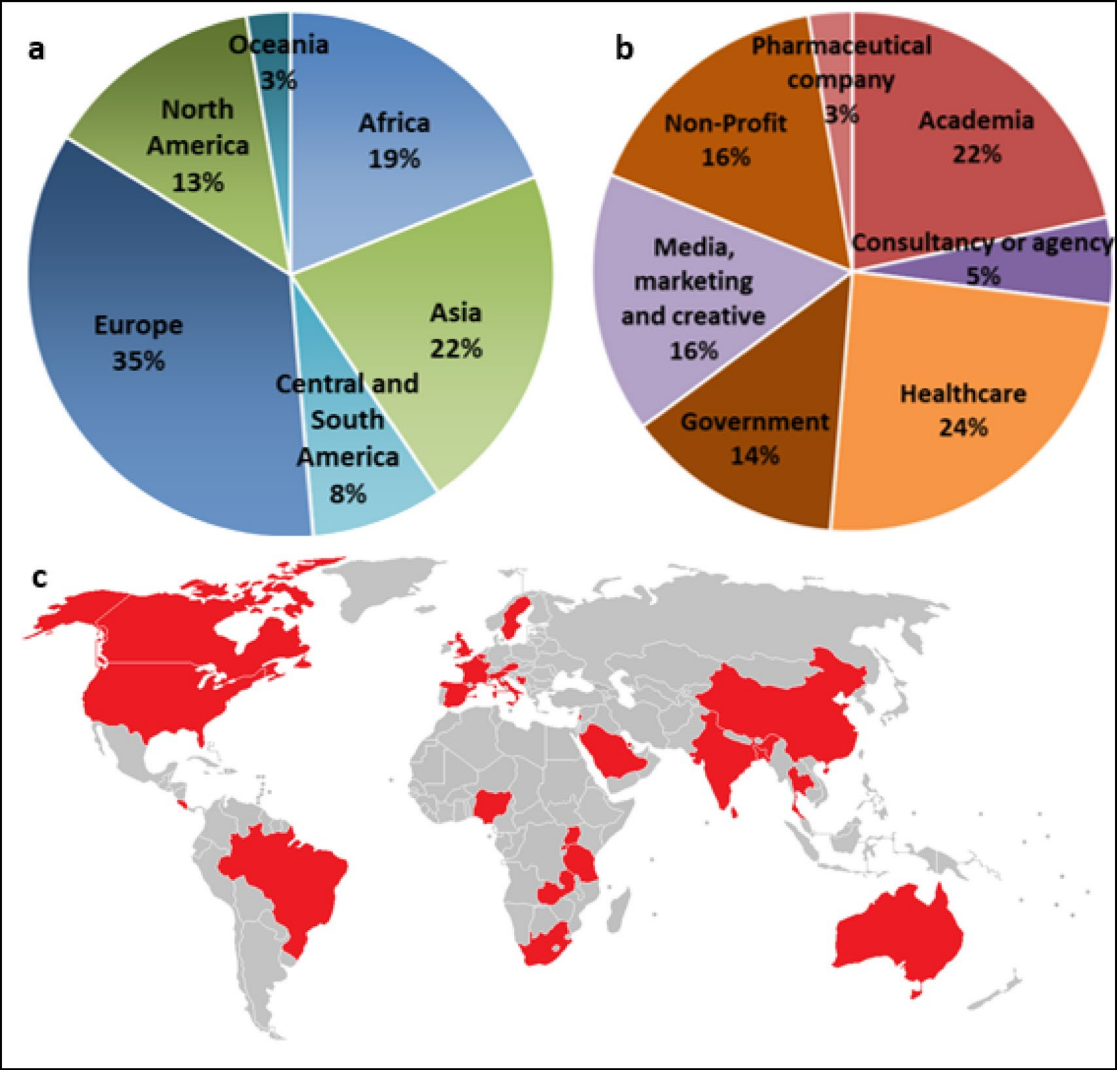
Key demographics of e-Delphi panellists. (**a**) Distribution of participants’ country of work by continent. (**b**) Distribution of participants’ employment sectors. **c** Participants’ work locations in the context of a global map.

35 experts were personally invited to participate in the e-Delphi study. Of these, 11 declined or did not reply, with two of these individuals suggesting replacements. Approximately 50 individuals got in touch following a study advert on social media, of which 12 fulfilled the study criteria and were invited to participate. The final e-Delphi panel consisted of 37 international experts in public-facing AMR communications. Participants had a mean of 10.5 years’ experience (*SD* = 7.29) in relevant communication, including 17 males and 20 females, with a mean age of 45 years (*SD* = 12.32). Participants worked across 27 different countries from five continents (Fig. 2) with 28 different nationalities and 24 native languages represented. Twenty-three participants self-identified as non-native English speakers. The majority of participants were employed in healthcare, academia, non-profit organisations, and media, marketing & creative industries (Fig. 2). Of the 37 panellists invited to the study, 100% completed Round 1, 36 (97%) completed Rounds 1 and 2, and 33 (89%) completed all three rounds. For detailed demographic data see Supplementary Note 1.

#### Consensus results

**Fig. 3:**
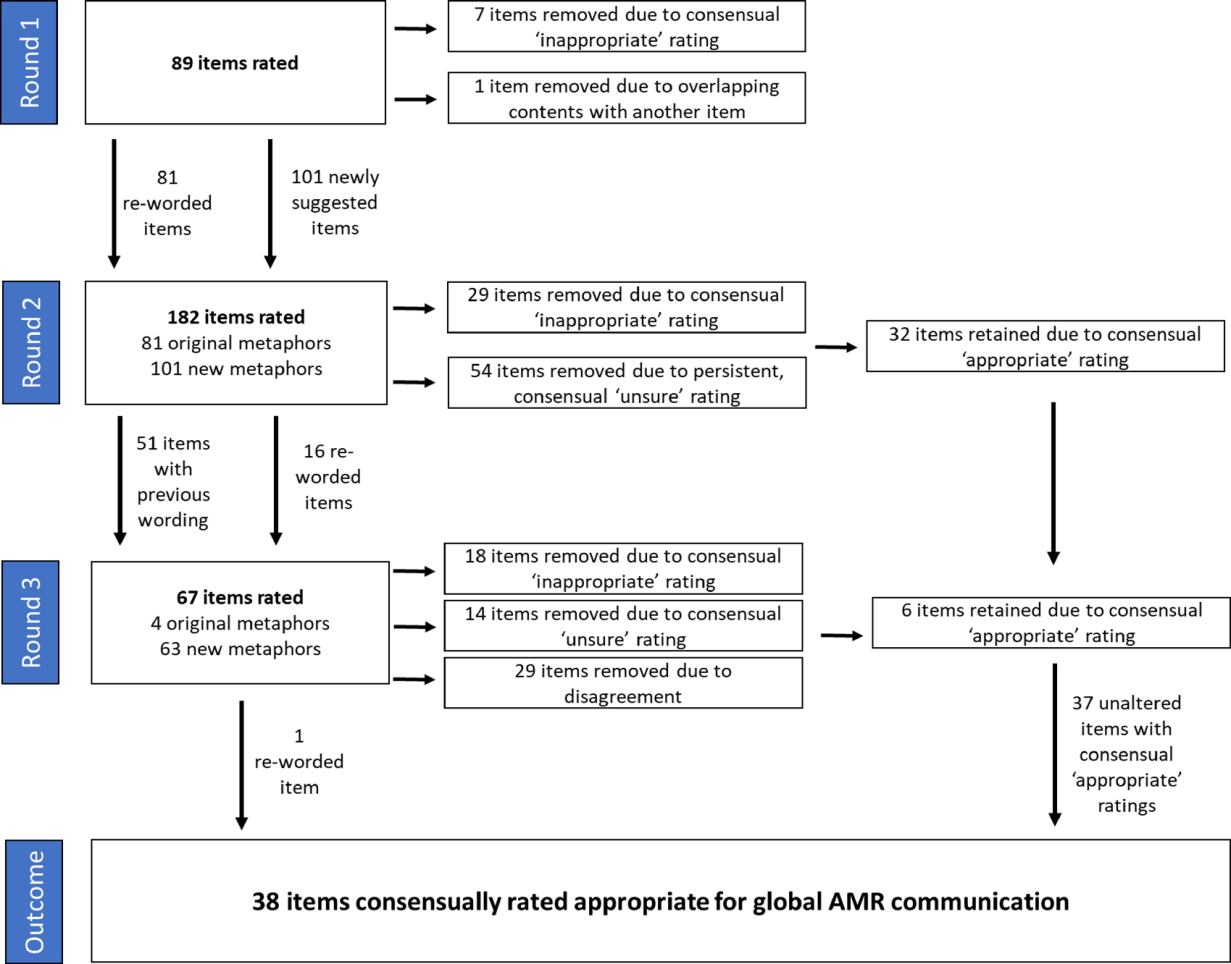
Overview of e-Delphi procedure and items rated in each round. Study process across three rounds of rating, including the number of metaphor items rated in each round, the origin of these items (original metaphor items suggested during co-design workshops or newly suggested metaphors from e-Delphi participants), the number of items removed in each round, and the number of items that were significantly reworded following participant feedback. All items from Round 1 were reworded following participant calls for shorter, more direct metaphors.

The process of our e-Delphi study with international expert AMR communicators is shown in Fig. 3. We conducted three study rounds, based on the aim to achieve consensus on a sufficient (i.e., useful) number of appropriate metaphor items. Considerations around usefulness were informed by the goal of offering multiple metaphor options for each misbelief or behaviour, enabling end-users to select the one they find most relatable in a given context.

Out of the 190 unique items rated across different rounds (see Supplementary Note 3), 161 (85%) achieved a panel consensus (i.e., no panel disagreement), with 38 (20%) rated appropriate (median rating ≥ 7), 59 (31%) rated inappropriate (median rating ≤ 3) and 64 (34%) rated unsure (median rating > 3 and 7< ). There were 29 items without clear consensus. Of these, only one item’s median was within the ‘appropriate’ range with a value of 7.

An overview of items that achieved a consensual final appropriateness rating of ≥ 7 is provided in Table 1, alongside section-specific summaries of qualitative feedback. An overview of all items rated during the e-Delphi is included in Supplementary Note 3.

**Table 1:** Median item ratings and disagreement across e-Delphi rounds for metaphors consensually rated ‘appropriate’ items rated in the e-Delphi exercise along with the median ratings of appropriateness (M) and whether there was disagreement among panellists in their rating (D). items are subdivided into thematic sections, with a brief overview of the section purpose and a summary of key qualitative feedback from participants. Only metaphor items that achieved a consensual appropriateness rating of ≥ 7 are included. “n/a” indicates that an item was not included in the respective round. items absent from round 1 were introduced in subsequent stages of the e-Delphi process. items excluded from round 3 had already reached consensus in earlier rounds.

Metaphor	Round 1		Round 2		Round 3	
	M	D	M	D	M	D
**Section 1: The microbial world is diverse** These metaphors tackle the misbelief that all microbes are the same. They lay the groundwork to understanding that antibiotics are not a solution to all infectious diseases, but only work against specific microbes. Participants thought effective metaphors should highlight both the beneficial and harmful roles of microbes, the risks of antibiotic misuse, and the fact that only bacteria respond to antibiotics. Participants cautioned against oversimplified or static comparisons that fail to capture microbial complexity and the seriousness of AMR.						
The world of germs is a garden full of diverse insects - some helpful, a few dangerous.	n/a	n/a	7	No	n/a	n/a
Communities of germs are like human neighbourhoods, made up of diverse residents including a few trouble-makers with the potential to cause chaos.	n/a	n/a	7	Yes	7	No
Germs are as varied as wild animals, living in balance but adapting to survive if threatened.	n/a	n/a	6.5	Yes	7	No
**Section 2: Bacteria can develop resistance**,** meaning that antibiotics lose effectiveness** These metaphors aim to explain the general concept of antibiotic resistance. Ideally, they also explain what causes resistance, thereby highlighting the link between antibiotic (over)use and resistance. Participants opposed belligerent war metaphors based on concerns around vilification of bacteria. Some participants worried that certain initial metaphor ideas (e.g., referencing bodybuilders or clever opponents) tended to frame resistance from the bacteria’s perspective, unintentionally casting them in a positive or admirable light. They warned this might undermine the seriousness of the threat or downplay the role of antibiotic misuse in driving resistance.						
Weeds resist weed killer—bacteria resist antibiotics.	7	No	7	No	n/a	n/a
Antibiotic-resistant bacteria are intruders wearing smart armour that upgrades itself when exposed to antibiotics.	6	No	6	No	7	No
Antibiotic-resistant bacteria change the locks to effective treatment, making our antibiotic keys useless.	n/a	n/a	7	No	n/a	n/a
Bacteria are like flowing water, finding creative ways around barriers.	n/a	n/a	7	No	n/a	n/a
**Section 3: Antibiotics only work against bacterial infections** These metaphors tackle the misbelief that antibiotics can be used to treat viruses. They thereby address problematic patient behaviours such as inappropriate demands for antibiotics or inappropriate self-medication. Participants emphasised that messaging should focus on the right to health rather than individual blame, and that metaphors must clearly convey the consequences of antibiotic misuse. While some personifying metaphors were perceived as engaging (e.g., antibiotics speaking the wrong language), tool-based comparisons were seen as more effective in illustrating the dangers of inappropriate use.						
Using antibiotics for viruses is using the wrong key to unlock a door.	7	No	8	No	n/a	n/a
Using antibiotics for viruses is like putting diesel in a petrol car.	7	No	7	No	n/a	n/a
Using antibiotics for viruses is like entering the wrong password for your username.	n/a	n/a	8	No	n/a	n/a
Using antibiotics for viruses is using the wrong charger for your phone.	n/a	n/a	8	No	n/a	n/a
Using antibiotics for viruses is putting earphones over your eyes.	n/a	n/a	6.5	Yes	7	No
Using antibiotics for viruses is changing the oil in your car when you have a flat tyre.	n/a	n/a	7	No	n/a	n/a
Using antibiotics for viruses is using salt to sweeten your coffee.	n/a	n/a	7	No	n/a	n/a
**Section 4: It’s the bacteria that develop resistance** These metaphors tackle the misbelief that the body becomes resistant. As such, they should explain that antibiotic resistance can spread and can affect individuals, who have never taken antibiotics in their life. This misbelief seemed the most difficult to address through metaphors. Participants noted that framing bacteria as simply “resistant” or “non-resistant” reflects a narrow clinical view and overlooks their complex, dual roles within the human microbiome. They also cautioned against metaphors that reinforce outdated notions of bacteria as entirely good or bad.						
Just like a bad apple spoils the whole bushel, a few resistant bacteria can affect the wider population—changing the balance and spreading resistance to others.	n/a	n/a	7	No	n/a	n/a
Like a single missing roof tile can leave a room drenched, one resistant bacterium can compromise the protection antibiotics offer.	n/a	n/a	7	No	n/a	n/a
**Section 5: Antibiotics should never be shared with other patients** These metaphors tackle misbeliefs that it is fine to share leftover medication with friends and family. This section initially contained many hygiene-based metaphor ideas. Participants thought these can help illustrate the risks of sharing antibiotics, but they may oversimplify the issue and miss key nuances - like patients reusing their own leftover medication. They emphasised that the real concern is whether antibiotics are appropriate and medically guided, not just whether they’re reused. Effective messaging should highlight the dangers of self-diagnosis and sharing, especially in low-resource settings, and clearly convey risks like allergic reactions, microbiome disruption, and resistance - without sounding dismissive or simplistic.						
Sharing leftover antibiotics is using your house key for someone else’s door.	n/a	n/a	7	No	n/a	n/a
**Section 6: You should complete your course of antibiotics as prescribed** These metaphors tackle misbeliefs that it is fine to stop taking antibiotics without consulting a qualified medical professional. Some participants noted that while stopping antibiotics early can leave infections untreated, the idea that it directly causes resistance is outdated. They emphasised that resistance results from overall antibiotic use, not just incomplete courses. Yet, other participants stressed the importance of following medical advice to avoid unresolved infections and spreading resistant strains. Metaphors should convey real risks and consequences, not just futility, and avoid outdated or fear-based messages.						
An unfinished course of antibiotics is a fire half-out. Sparks remain, ready to flare up again even stronger.	7	No	8	No	n/a	n/a
An unfinished course of antibiotics is a partially weeded garden with roots left behind that will grow back even more resilient.	7	No	7	No	n/a	n/a
An unfinished course of antibiotics is a half-constructed house with no roof.	n/a	n/a	7	No	n/a	n/a
An unfinished course of antibiotics is leaving the shelter before the storm has passed.	n/a	n/a	7	No	n/a	n/a
An unfinished course of antibiotics is turning off the stove before the food is fully cooked.	n/a	n/a	7	No	n/a	n/a
An unfinished course of antibiotics is a half-built bridge.	n/a	n/a	7	No	n/a	n/a
**Section 7: Patients need to follow their doctors’ advice** These metaphors tackle the misbelief that self-diagnosis is a sufficient basis for decision making about antibiotics. They address associated problematic behaviours of purchasing antibiotics via questionable sources or pressurising doctors for prescriptions. Participants criticised initial metaphor ideas that blamed patients for antibiotic misuse as condescending and unrealistic, especially in contexts with limited healthcare access and declining trust in medical systems. They emphasised the need for culturally inclusive, emotionally resonant messaging that reflects real-life complexities and portrays patients as informed decision-makers. Effective metaphors should focus on practical consequences and promote collaborative, respectful relationships between patients and providers.						
Ignoring medical advice is baking without a recipe.	7	No	7	No	n/a	n/a
Ignoring medical advice is trying to build a rocket without an engineer.	n/a	n/a	6	No	7	No
Ignoring medical advice is sailing into open waters without a captain or a compass.	n/a	n/a	7	No	n/a	n/a
Ignoring medical advice is flying a plane without training.	n/a	n/a	7.5	No	n/a	n/a
Ignoring medical advice is climbing a dangerous mountain without a guide.	n/a	n/a	4.5	Yes	7	No
**Section 8: Antibiotics aren’t always required for minor infections** These metaphors tackle the misbelief that all bacterial infections require antibiotic treatment, thereby reducing antibiotic expectations. Participants found vivid metaphors effective when they clearly conveyed risk or waste, but criticised clichéd or violent imagery as condescending. They stressed that impactful metaphors should show both overreaction and harm. Additional explanation may be required to explain the term “minor infection”.						
Using antibiotics for minor infections is using a sledgehammer to kill a fly.	7	No	7	No	n/a	n/a
Using antibiotics for minor infections is using a hammer to crack a peanut.	7	No	7	No	n/a	n/a
Using antibiotics for minor infections is using a fire extinguisher to blow out a candle.	7	No	7.5	No	n/a	n/a
Using antibiotics for minor infections is like spraying your whole garden with weed killer to remove a couple of plants.	n/a	n/a	7	No	n/a	n/a
Using antibiotics for minor infections is visiting a surgeon for a scratch.	n/a	n/a	7	No	n/a	n/a
**Section 9: Infection prevention helps to avoid antibiotic use** These metaphors highlight the links between hygiene, infection control, vaccination and AMR, which many lay audiences may not be aware of. They should demonstrate the value of behaviours to prevent infection as a step towards avoiding antibiotic use and curbing AMR. Some participants felt that infection prevention metaphors tied to AMR can be overly complex and less motivating than messages focused on immediate health risks. They recommended simpler, relatable metaphors - like everyday habits - that highlight how prevention reduces antibiotic use and promote stewardship through shared responsibility. Messages should be clear, positive, and grounded in familiar behaviours to encourage real change.						
We can save our antibiotics by avoiding infection just like we avoid emergency services by maintaining our car.	6	No	7	No	n/a	n/a
We can save antibiotics by avoiding infection, just like we avoid endless mopping by fixing a leaky pipe.	n/a	n/a	7	No	n/a	n/a
We can save antibiotics by avoiding infection, just like we can save firefighters by preventing outbreak of fire.	n/a	n/a	7	No	n/a	n/a
We can save antibiotics by avoiding infection, just like we avoid cavities through brushing our teeth.	n/a	n/a	7	No	n/a	n/a
We can save antibiotics by avoiding infection, just like we protect ourselves from accidents by wearing seatbelts.	n/a	n/a	7.5	No	n/a	n/a

#### General considerations shaping metaphor ratings

Participants’ free-text comments revealed a strong preference for short, direct metaphorical mappings, rather than extended metaphors, analogies, or similes. Simplicity and clarity were seen as key to create vivid mental images. Metaphors grounded in relatable, everyday experiences were especially well received, particularly those that avoided complex or obscure language (e.g., references to flora and fauna, see item 1c in Supplementary Note 3) and could be easily understood across diverse contexts.

Concerns about generalisability led to the rejection of culturally-specific metaphors. Examples such as dominoes (item 3m), kaleidoscopes (item 1h), dodgeball (item N2.4), orchestras (item 1f), and religious references (e.g., to the biblical figures of Samson and Delilah, item N2.5) were seen as too niche or context-dependent. Similarly, metaphors referencing geographically specific extreme weather events like warm winter coats (item 2k) or hurricane control (item N8.5) were considered problematic. Participants also warned that certain metaphors—such as those based on video gaming (e.g., item 3n)—could alienate specific demographic groups, particularly older adults. Geographic variations in terminology, such as “football” vs. “soccer” or “petrol” vs. “gas,” were flagged as potential sources of confusion.

There was also concern about metaphors that could touch on controversial or sensitive topics. Socially sensitive references (e.g., descriptions of bacteria as *rebellious teenagers*, item N2.11) were highlighted as potentially stigmatising. Metaphors that might be culturally inappropriate or offensive to some (e.g., women using urinals, item 3h) or those considered vulgar (e.g., references to personal hygiene products, items 5a-c), were discouraged.

A key challenge identified was the balance between conveying threat or urgency while maintaining nuance—especially when discussing the symbiotic and essential roles of bacteria in human life. Participants warned against both extremes: apocalyptic scaremongering and overly romanticised portrayals of human-bacteria relationships (e.g., references to dance and balance, item N1.5). Participants also questioned whether simple metaphors could adequately capture the complexity of the information being communicated. In some cases, they suggested that metaphors may need to be embedded within additional explanatory content to provide sufficient context.

One particularly debated topic was the use of war metaphors. Most participants opposed belligerent language, arguing that it evoked aggression, was overused, and oversimplified the nature of bacteria by casting them as hostile enemies. Such metaphors were also seen as potentially alienating, especially for individuals with personal experiences of war. Sports metaphors were similarly rejected in some cases, as they could imply rivalry and conquest, echoing the combative tone of war metaphors. However, a few participants acknowledged that despite their drawbacks, war and combat metaphors are often easily understood by the public. To retain a sense of urgency without invoking warfare, some suggested alternative frames—such as sci-fi battle scenarios—that could convey conflict in a more abstract and less emotionally charged way.

## Discussion

This study presents the first systematic, theory-driven approach to metaphor co-creation to enhance global AMR risk communication and promote behaviour change. Through a series of stakeholder co-design workshops and an international e-Delphi study with expert AMR communicators, we arrived at a set of 38 recommended metaphors appropriate for global public health messaging. All newly generated metaphors can be accessed in Supplementary Note 4 and may be included in future risk communication efforts about AMR, under CC BY 4.0 license.

Metaphor source domains drew on a range of topics, but nature/gardening emerged as one of the most endorsed domains, most likely due to shared universal experiences of our natural environment. Examples include likening the microbial world to a *garden full of diverse insects*, comparing antibiotic-resistant bacteria to *weeds resisting weed killer* or *flowing water finding creative ways around barriers*, and likening incomplete antibiotic treatment courses to a *partially weeded garden where roots left behind will grow back even more resilient*. Nature metaphors have appeared in other health research. For example, cancer patients found meaning in nature-based symbolism, drawing insights from *natural life cycles*^46^, or conceptualising their illness as *garden overgrown with weeds*^47^. These garden and nature metaphors represent a fresh perspective within the AMR discourse, diverging sharply from the dominant war and battle imagery found in earlier literature^33–35,48^.

Tools/engineering provided another popular metaphorical source domain, with particular emphasis on lock-and-key mechanisms, car maintenance, and household tools This is likely due to the simplicity and relatability of these concepts. To explain the inappropriate use of antibiotics for viral infections, for example, metaphors described *using the wrong key to unlock a door*, *entering the wrong password for your username*, or *putting diesel in a petrol (gas) car*. Excessive use of antibiotics was compared to using a *sledgehammer to kill a fly* or a *hammer to crack a peanut.* Tool and engineering references have been scarce in previous health and AMR messaging, with the exception of references to biological cell mechanisms through images of *pumps* and *blueprints*^33^, and occasional mention of machinery, for example descriptions of healthcare workers as *cogs in the system*^49^. Less emotionally charged than earlier AMR metaphors—typically framed around existential threat—e-Delphi participants appreciated these for their apt conceptual mappings and grounded, everyday relevance.

One metaphor domain that generated fewer items but particularly high appropriateness ratings was fire fighting. An unfinished course of antibiotics was compared to a *fire half-out*,* with sparks remaining*,* ready to flare up again even stronger*. Antibiotic overuse was likened to using a *fire extinguisher to blow out a candle*, and infection prevention was conceptualised as *saving firefighters by preventing outbreak of fire*. Firefighting metaphors have previously appeared in discussions of infectious diseases, particularly during the COVID-19 pandemic^50^, and have also occasionally surfaced in public discourse on AMR^51^—for example, through a hashtag in the phrase “Antibiotics are the #FireExtinguishersOfMedicine” on the expert blog “AMR.Solutions”^52^. Compared to metaphors drawn from nature or tools, firefighting imagery may offer the added benefit of conveying emotional intensity and urgency^53^, while avoiding overly dramatised sci-fi narratives such as “Drugmageddon” or apocalyptic visions of a return to the dark ages^33^.

Interestingly, even though our co-design workshops with UK members of the public and doctors generated a large number of war metaphors—for example describing microbes as *army of microscopic warriors*, antibiotic-resistant bacteria as *soldiers*,* who have learned the enemy’s tactics*, and AMR as *fortress that protects bacteria from antibiotic treatment*—none of these metaphors received high ratings by the e-Delphi panellists. The only endorsed metaphor that evoked combat was the comparison of antibiotic-resistant bacteria to *intruders wearing smart armour that upgrades itself when exposed to antibiotics*. Indeed, most expert AMR communicators expressed strong opposition to war metaphors and even sporting metaphors (e.g., describing antibiotic-resistant bacteria as *players from opposing teams*), which were perceived by some as carrying undertones of conflict and battle. This echoes theoretical critiques that such framings oversimplify complex biological processes into binary narratives of good versus evil and may inadvertently assign blame^33^, and previous work cautioning against the use of militaristic language in health communication^45,50^. Yet our findings highlight a possible disconnect between expert preferences and what actually resonates with certain populations—in this case the UK public and doctors. Indeed, some e-Delphi panellists noted they disliked war imagery but sometimes felt compelled to use it as it best conveyed urgency to lay populations. The lasting appeal of war rhetoric may also stem from its familiarity and the ease with which it is processed, developed through years of conventional use^54^. Consequently, effectiveness of war metaphors may depend on context and culture—particularly where a sense of risk and emotional appeal are needed to drive behaviour change^55,56^. An additional consideration is that, while war metaphors may be effective in capturing attention and galvanising initial action, they could be less suited for conveying complex or explanatory content— where alternative metaphorical frames may facilitate more apt conceptual mappings^56^.

Of all the misbeliefs tackled through metaphor in the present study, antiviral efficacy emerged as the one most commonly addressed, with a total of seven targeted metaphors. It is likely that the key aspects communicated within these metaphors—futility, potential harm, and wasted resources—are particularly relatable, thereby making it easier to create apt metaphorical mappings. By contrast, only a few metaphors were deemed appropriate to target the misbelief that bacteria, rather than the human body, develop resistance, or that antibiotics should not be shared with others. The first concept may be too technical or abstract, making it difficult to find direct analogy in everyday experience. The theme of inappropriate sharing, while more relatable, was controversial due to participant concerns regarding condescending and culturally controversial comparisons. This highlights distinct challenges in developing new metaphors, and potential contexts, where the potential of figurative speech may be limited.

Notably, the final list of metaphors varied in how well items captured different aspects of each misbelief or problem. The first theme concerning microbial diversity aimed to bridge knowledge gaps regarding the various types of microbes, thereby establishing a foundational understanding that antibiotics are effective solely against bacteria. However, the metaphors recommended leaned more towards conveying the multifaceted roles of microbes, emphasising that pathogenicity is both selective and context-dependent. Another example is the *flowing water* metaphor, which encapsulates the adaptive, creative and unstoppable nature of microbes, but is less successful in explaining exact mechanisms of resistance. Similarly, while *lock-and-key* metaphors highlight the inappropriateness of antibiotic use for viral illnesses, they are limited in their ability to communicate harm and damage caused by misuse. Having a menu of 38 endorsed metaphors to choose from means that messages can be purposefully selected depending on the specific aspect of AMR that communicators aim to draw attention to. Likewise, metaphors drawn from related source domains could be combined to convey multiple dimensions of AMR and construct a more comprehensive narrative.

Notably, while carefully designed and refined with different stakeholders and communication experts, the recommended metaphors have not empirically been tested for their ability to engage, raise awareness, communicate knowledge, change attitudes or motivate behaviour change. Future opportunities lie in testing and comparing the effectiveness of different metaphorical AMR messages, for example through pre- and post-exposure attitude assessment or forced-choice tasks^57^.

Beyond the specific metaphor recommendations, this study yielded broader insights into challenges and opportunities for AMR communication. Free-text comments from e-Delphi participants illuminated the rationale behind metaphor selection, revealing a preference for short, simple, and clear messages grounded in everyday experience. It also highlighted a nuanced effort to balance the portrayal of threat with the recognition of bacteria as essential and life-sustaining organisms. This tension reflects longstanding communication dilemmas: how to foster urgency and motivation without inducing alarmism or panic^58^.

A further communication challenge identified by the e-Delphi experts was the inherent complexity of AMR as a topic relative to other public health issues. Effective messaging must find a careful compromise between conveying detailed, evidence-based information and maintaining clarity and accessibility for diverse audiences. This tension is particularly pronounced in areas of scientific uncertainty, such as the relationship between antibiotic use and AMR risk. Panel discussions around metaphors for self-directed termination of antibiotic courses revealed divergent views, with some experts cautioning that optimal treatment durations remain unclear and that shorter courses may, in some cases, be preferable^41,42^. These findings highlight another potential limitation of metaphorical messaging in capturing scientific ambiguity and suggest that additional information may be required to communicate evolving evidence responsibly.

Finally, the international composition of the expert panel underscored the difficulty of crafting universally resonant messages. Participants highlighted the need to account for variation in demographic profiles, cultural contexts, geographic experiences, healthcare access, and education levels including literacy—this applies to both the selection of metaphor domain as well as the selection of the particular AMR aspects global communicators should address. While the metaphors developed in this study were considered appropriate for global dissemination, the findings suggest that context-specific adaptations may be necessary to address specific local beliefs, behaviours and challenges within the targeted demographic groups. Particular attention was drawn to low and middle income (LMIC) contexts, where medicine sharing and self-diagnosis may be pragmatic behaviours developed to manage medication shortages and difficulties in accessing advice from qualified healthcare professionals. This was corroborated through insights from the co-design workshop in South Africa, where metaphor ideation drew heavily on regional experiences and idioms, for example making reference to precarious housing. While few of these metaphors resonated with the international e-Delphi panel, opportunities lie in integrating locally rooted metaphors—expressed in native languages—into targeted communication campaigns, particularly in cultures where storytelling plays a central role in communication^59^. To this end, the study protocol and materials offer a replicable framework for co-producing culturally, demographically and geographically tailored AMR messages. The materials can also support public engagement workshops that elicit context‑specific preferences for particular metaphors from the list of 38 recommendations, enabling local communicators to make informed choices about which messages to incorporate into their campaigns.

This study has a few limitations. The initial co-design workshops in the UK and South Africa had a comparatively narrow geographic focus and varied in format due to practical and funding constraints. The selection of one high-income country from the Global North and one middle-income country from the Global South was a deliberate strategy to balance cultural and epidemiological diversity within available resources. In the UK, we conducted a series of small, primarily virtual workshops which included both doctors and members of the public in separate sessions. By contrast, in South Africa, we held a single larger in-person workshop with only members of the public. Format differences reflected local recruitment strategies, participant preferences, and ethical requirements. Although a uniform approach might have supported methodological consistency, the flexible design allowed us to respond to local contexts and maximise creative potential.

Another limitation is the exclusive focus on English-language metaphors. Although co-design stakeholders and e-Delphi participants came from diverse linguistic backgrounds, all study outputs were produced in English. Since metaphors are closely tied to language and culture, it remains uncertain whether those generated in this study can be effectively translated into other languages. Future opportunities lie in developing and testing translations across different contexts.

Finally, the study focus was limited to human health. While some of the metaphors generated—particularly those regarding microbial diversity and mechanisms of AMR—might translate to other areas of One Health (animal and environmental health), metaphors relating to specific patient behaviours will not apply to antibiotic choices in agriculture, veterinary practices or wastewater treatment. Future work is necessary to gain additional insights into effective messaging strategies across different stakeholders of the One Health umbrella.

To conclude, this study reports the first systematic effort to co-design novel and apt metaphors for improving global AMR communication. We developed 38 expert-approved metaphors targeting common misconceptions and problematic behaviours. Drawing from domains like nature, gardening, tools, engineering, and firefighting these metaphors avoid alarmist war imagery and instead use relatable, everyday symbolism that resonates across cultures and regions. Future AMR communications could incorporate these metaphors or adopt the study’s methodology of co-design and consensus-building to prioritise specific information or create additional messages addressing local needs. Challenges will remain in striking a delicate balance—conveying the urgency of the issue without inciting panic, and translating complex scientific insights into clear, practical guidance.

## Method

Ethical approval for the UK co-design workshops was obtained from the NHS England Health Research Authority and Health and Care Research Wales on 1 August 2024 [IRAS project ID: 341826, REC reference: 24/SW/0082]. Ethical approval for the South African co-design workshop was obtained from the University of Cape Town Human Research Ethics Committee [HREC REF: 692/2023] on 22 May 2024. Ethical approval for the international e-Delphi study was obtained from the University of Leicester Medical and Biological Sciences Research Ethics Committee on 7 April 2025 [Project ID: 2992]. All procedures adhered to the ethical standards and regulatory requirements set forth by the respective ethics committees. Written informed consent was obtained from each participant before their enrolment in the study. A prospective research protocol detailing the full methods plans was published prior to the commencement of the second e-Delphi round in May 2025 and can be accessed via the Open Science Framework: https://osf.io/r4tqx/.

### Co-design

#### Design

To generate initial metaphor ideas for the first round of the e-Delphi study, we conducted co-design workshops in the UK and South Africa. We used the definition of co-design as “active collaboration among stakeholders relating to solution design, given a pre-determined problem”^60^, employing a design-based rather than social science-based approach that focused on brainstorming and reflection^61^. Our method was informed by social innovation theory, which is concerned with the way that new ideas, based on collaboration and empowerment, can address social challenges in more effective and inclusive ways compared to existing solutions^62^. Our preparation of materials to support the ideation process were additionally informed by conceptual metaphor theory^23^, which emphasises the importance of familiar and uncontroversial metaphorical source domains due to their cognitive accessibility and easy translation across different contexts. Additionally, we considered the importance of different metaphor dimensions, most importantly that of aptness, which refers to how well metaphors capture essential aspects of the problem^63^. Finally, the metaphor generation prompts were systematically aligned with prevalent misconceptions and behavioural challenges documented in prior research and public knowledge surveys, as referenced in the Introduction. Particular attention was given to the widespread misbeliefs that antibiotics are effective against viral infections and that resistance develops within the human body rather than bacteria, reflecting their persistent recurrence in earlier studies.

#### Participants

Participants were recruited from the UK and a South African township characterised by a mix of formal housing and extensive informal settlements located in the Western Cape province (Khayelitsha). Countries and locations were selected to reflect varied perspectives across the Global North and South, encompassing diverse contexts in terms of disease burden, healthcare governance, and culture. Instead of aiming for representative samples, opportunity sampling was used to capture distinctive viewpoints. In the UK, we ran several small workshops with hospital doctors and members of the public with recent experience of antibiotic-resistant infections between November 2024 and February 2025. UK participants were recruited via social media advertising (X, Bluesky, LinkedIn and Facebook), invitation emails sent to professional contacts and networks of hospitals doctors, and invitation letters sent to members of the public with recent hospitalisation for an antibiotic-resistant infection, identified via hospital records at University Hospital of Leicester NHS trust. In South Africa, we conducted one larger co-design workshop on 1st April 2025 with members of the public, who had experience and knowledge of infection. The recruitment strategy involved an email invitation to registered participants and followers of the community and public engagement non-profit organisation Eh!woza, located in the township Khayelitsha on the outskirts of Cape Town, Western Cape. Over half of Khayelitsha’s predominantly Black African population live in informal housing, and the area faces extreme poverty and unemployment (estimated at 38.3%). It also has some of South Africa’s worst health indicators, including high rates of tuberculosis (37%) and HIV/TB co-infection (47.6%)^64^.

#### Materials and procedure

The co-design process was embedded within wider research workshops. In the UK, they started with a series of semi-structured questions about previous experiences of antibiotic-resistant infections reported separately^18^, in South Africa they began with a reflection on recent experiences of infection. Then the workshops proceeded to an interactive brainstorming session. The ideation process was pre-empted by a short presentation that provided background on AMR, the concept of metaphor, and common knowledge gaps—with a specific focus on antibiotic use for viruses and the unit of resistance (see Supplementary Note 5). Participants were shown examples of metaphor use in health and practiced metaphor ideation on the simpler example of self-care when suffering from a cold. Then, participants were guided through a series of creative prompts and ideation techniques adapted to the group and setting (e.g., breakout groups, mind-mapping, note-writing and rating the winning metaphor idea). Once metaphor ideation around the misbeliefs that antibiotics can treat viral infections and that the body, rather than bacteria, becomes resistant had tapered off, the researchers introduced additional prompts about common patient behaviours that contribute to antibiotic overuse, such as self‑medication, sharing antibiotics, and non‑adherence to treatment advice. Participants were free to explore any aspect of antibiotic resistance during the ideation process, although most suggestions ultimately aligned with the prompts provided. In the UK, participant preferences meant that all but one workshop were conducted online. Each UK workshop was led by one researcher (EK) and all workshops with members of the public were additionally co-facilitated by a creative strategist, using software such as Miro boards and MS Whiteboard to capture ideas during the sessions. Workshops were sequential, meaning that some metaphors from previous workshops were fed into subsequent workshops to stimulate further ideas and allow for discussion. UK workshop duration was approximately 90–120 min for members of the public and 30 min for doctors. In South Africa, one 2-hour workshop was held face-to-face, co-facilitated by SM and AK, both researchers with experience in public engagement and science communication. The workshop was held in English, but SM offered interpretation into isiXhosa where required. In those instances, where participants’ group discussions and brainstorming took place in isiXhosa, SM provided a summary of discussion outcomes in English. Participants were invited to submit additional metaphor ideas after conclusion of the workshops.

#### Analysis

Formal data collected included (1) visual outputs such as electronic comments and mind maps via Miro boards and MS Whiteboard, (2) written notes from researchers and participants during the workshops, (3) metaphor ideas submitted by participants post-workshop, and (4) anonymised transcripts of audio-recordings (with translation from isiXhosa into English in the case of the South African workshop). No formal qualitative analysis was performed, because the resulting data was dispersed across multiple media and sources and often lacked the accompanying dialogue or justification needed for more in‑depth approaches, such as reflexive thematic analysis. Instead, all metaphor ideas were captured verbatim and subsequently reviewed by the research team to check for accurate theoretical mappings, to refine wording, remove duplicate ideas and thematically categorise by the misbelief or behavioural challenge they aimed to address. This thematic categorisation was largely informed by existing literature on common knowledge gaps. It incorporated two well‑established misbeliefs—the idea that antibiotics can treat viral infections and the assumption that the body, rather than bacteria, becomes resistant—which had been explored in depth at the start of the co‑design workshops. It also drew on participants’ reflections about problematic patient behaviours that were introduced once initial discussion had tapered off (e.g., self‑medication, sharing antibiotics, overuse, and non‑adherence to treatment advice). Not all themes originated from researcher prompts, however. Clinician participants raised the importance of infection prevention, which was subsequently developed into a separate thematic category. The resulting, structured list of metaphor ideas was entered into the next stage of the study, the e-Delphi consensus procedure. Our analysis drew on a critical realist framework, which acknowledges the role of socio-cultural factors in shaping perceptions of reality^65^. The lead researcher engaged in ongoing reflexivity to address potential biases when selecting and refining metaphors for the e-Delphi, including personal inclinations toward certain metaphors and prior critiques of war-related imagery^33^. A research diary with reflexive annotations was maintained to reduce bias.

### e-Delphi

#### Design

The e-Delphi consensus method offers a structured and iterative way to engage subject-matter experts in developing and refining practical solutions to complex health issues^66^. It has been used successfully to develop communication strategies in other contexts, including medicines risk^67^, oncology^68^, multi-level health communication^69^, and the use of AI in public health messaging^70^. The process included multiple online study rounds to: (1) evaluate agreement with the initial metaphor list obtained through co-design; (2) gather suggestions and clarifications to extend and refine it; and (3) determine which items are deemed appropriate for global risk communication about AMR. The RAND/UCLA Appropriateness method^71^was used to assess appropriateness and disagreement. Although reporting checklists for Delphi studies exist, some adopt terminology and assumptions drawn from survey methodology, such as sampling-based inference, or encourage specifying the number of rounds in advance, which do not reflect the way we approach Delphi as a consensus method. We therefore report our procedures in line with core Delphi principles rather than adhering to a specific checklist.

#### Participants

For the purpose of the e-Delphi study, AMR communication experts were defined as individuals with at least 3 years’ experience communicating about AMR with the general public, who are “…representative of their profession, have power to implement the findings, or because they are not likely to be challenged as experts in the field”^72^. We anticipated attracting 30–40 experts that fitted this definition, from across a wide range of geographical locations and backgrounds. The lead author accessed existing professional networks and purposefully identified global AMR communication experts via web pages of relevant international organisations, published conference panels or social media networks, who were personally invited to participate. Additionally, one advert was placed on the lead author’s LinkedIn page.

#### Materials and procedure

A custom-built online platform by Clinvivo Ltd (https://www.clinvivo.com/) was used to host the web-accessed e-Delphi questionnaires. The online environment allowed experts to rate items and provide anonymous feedback, as well as summarising panel aggregate ratings and views back to them in subsequent rounds, along with their own prior ratings. Clinvivo sent participation reminders to minimise panel attrition throughout the e-Delphi process. Round 1 ran from 22 April – 06 May, Round 2 from 29 May – 23 June and Round 3 from 10 July – 5 August 2025.

The first round of the study began with a demographic questionnaire. Then, participants were presented with a set of introductory materials that involved background information on AMR risk communication and metaphor theory, as well as example rating exercises of metaphors (see Supplementary Note 5). Participants were subsequently asked to rate the initial metaphors that evolved from the co-design process on the dimension of appropriateness, ranging from 1 = highly inappropriate to 9 = highly appropriate. When judging appropriateness, participants were asked to consider the following aspects:

- Aptness (capturing and communicating specific aspects of AMR),
- Universality (familiarity and relatability irrespective of culture or upbringing).
- Persuasiveness (challenging misunderstandings and encourage appropriate action).

The metaphors for rating were presented in sections that grouped similar metaphors together. Sections were again based around particular misbeliefs or problematic behaviours that the respective metaphors aimed to address (e.g., the misbelief that antibiotics can treat viruses or the problematic behaviour of sharing leftover antibiotics). Participants were encouraged to offer their own metaphor ideas for each section or provide open comments. Several section themes were refined in response to qualitative feedback from participants. Notably, Section 7—originally centred on self-diagnosis—was revised following input from e-Delphi experts, who identified this framing as potentially problematic in LMIC contexts, where access to qualified healthcare professionals may be limited and self-directed treatment is often a pragmatic necessity. Consequently, panellists recommended reframing the section to address intentional non-adherence, thereby better capturing the behavioural dynamics relevant across diverse healthcare settings. The questionnaire allowed for completion in multiple sittings, with ratings editable before final upload.

In the subsequent e-Delphi rounds, the rating process was replicated for newly suggested items. For items that were rated in the previous rounds, participants received summaries of other participants’ comments, details of the panel aggregate, their own prior rating, and a request to rerate the item following consideration of fellow experts’ views. Free-text boxes were once again provided for each section, so participants could add suggestions for improvements.

#### Data analysis

Following each round, descriptive statistics were used to summarise the participants’ ratings of appropriateness. The appropriateness ratings were also analysed using the RAND/UCLA Appropriateness method^71^. Within this, the median rating score for each metaphor item was calculated. A median within the ranges of 1–3, 4–6 or 7–9 of the rating scale was judged to reflect inappropriateness, uncertainty, and appropriateness, respectively. The inter-percentile range (p30, p70; IPR) was then calculated to determine the spread of ratings for each item. A disagreement index was subsequently calculated based in the IPR adjusted for asymmetry of ratings^71^. A disagreement index greater than 1 was interpreted as presence of panel disagreement. Metaphors were excluded from the next round if rated either inappropriate (median score of ≤ 3) or appropriate (median score of ≥ 7), without disagreement (disagreement index ≤ 1). Items rated appropriate were retained for another round if the items’ wording was changed significantly following participant comments.

Qualitative comments, including suggestions for new items and modifications to existing wordings were collated by the research team. Summaries of panellists’ comments with typifying examples, in particular from participants with high or low ratings for an item, were produced to provide feedback to the participants in the next round, as appropriate. Any additional metaphor ideas were screened for duplication and where appropriate (e.g., in the case of typos or grammatical errors) reworded, while retaining semantic equivalence, for consistency with other metaphor items. Large language models (MS Copilot) were used to help refine wording in some instances.

## Supplementary Information

Below is the link to the electronic supplementary material.

Supplementary Material 1

Supplementary Material 2

## Data Availability

The quantitative data set from all three rounds of the e-Delphi study and the full list of all metaphor ideas across the different project stages can be accessed via the Open Science Framework: https:/osf.io/r4tqx.
