# Supplementary material for: Developing public health risk messages about antibiotic resistance using metaphors: an international co-design and e-Delphi consensus study
Source: Sci Rep. 2026 Feb 18;16:9788. doi: 10.1038/s41598-026-40577-5 (PMC13013596; doi:10.1038/s41598-026-40577-5)
Supplement: Supplementary file 2 — Supplementary Material 2 [file 41598_2026_40577_MOESM2_ESM.pdf]

Supplementary Information

for

Developing public health risk messages about antibiotic resistance using metaphors: An international co-design and e-Delphi consensus study

Eva M. Krockow[1*], Meghann Jones[1], Samkele Mkumbuzi[2], Marc Mendelson[2], Carolyn Tarrant[3,4], Robert Froud[5,6], Anastasia Koch[7,8], Stephen J. Flusberg[9], & Emma Pitchforth[10]

[1]*School of Psychology and Vision Sciences, University of Leicester, Leicester, United Kingdom*

[2]*Division of Infectious Diseases & HIV Medicine, Department of Medicine, Groote Schuur Hospital, University of Cape Town, Cape Town, South Africa*

[3]*School of Medical Sciences, University of Leicester, Leicester, United Kingdom*

[4]*National Institute for Health and Care Research (NIHR) Greater Manchester Patient Safety Research Collaboration (GM PSRC)*

[5]*School of Health Sciences, Kristiania University of Applied Sciences, Oslo, Norway*

[6]*Warwick Medical School, University of Warwick, United Kingdom*

[7]*Eh!woza, Khayelitsha, South Africa*

[8]*Molecular Mycobacteriology Research Unit, Institute of Infectious Disease and Molecular Medicine and Department of Pathology, University of Cape Town, Cape Town, South Africa*

[9]*Department of Cognitive Science, Vassar College, Poughkeepsie, NY, USA*

[10]*Faculty of Health and Life Sciences, University of Exeter, Exeter, United Kingdom*

Table of Contents

Supplementary Note 1: Detailed demographic participant data 3
Co-design 3
e-Delphi 4
Supplementary Note 2: Metaphors that emerged from co-design workshops 6
Supplementary Note 3: Metaphors rated across the three e-Delphi rounds 11
Original metaphor ratings 11
New metaphor ratings 26
Supplementary Note 4: Metaphors consensually rated 'appropriate' by the e-Delphi expert panel 36
Supplementary Note 5: Research materials 38
Co-design 38
UK Co-design Workshops: Introductory presentation slides 38
South African Co-design Workshop: Introductory presentation slides 45
e-Delphi 51
Background information materials for e-Delphi study 51
Instructions for e-Delphi Round 1 54
Instructions for e-Delphi Round 2 55
Instructions for e-Delphi Round 3 56
Example of e-Delphi item rating on Clinvivo Platform 58

Supplementary Note 1: Detailed demographic participant data

Co-design

Supplementary Table 1 Aggregate Demographic Data of Public and Doctor Participants in UK Co-Design Workshops

	**Public participants (*n*=15)**	**Doctor participants (*n*=14)**
Gender	Male = 5<br>Female = 10	Male = 8<br>Female = 6
Age	Mean = 46.5 years<br>SD = 19.85<br>Range = 21-74 years<br>Prefer not to say = 1	Mean = 43.2 years<br>SD = 13.5<br>Range = 23-64 years<br>Prefer not to say = 1
Ethnicity	Asian or Asian British = 5<br>Black, African, Caribbean or Black British = 5<br>White = 4<br>Prefer not to say = 1	White = 8<br>Asian or Asian British = 4<br>Black, African, Caribbean or Black British = 1<br>Other (Arab) = 1
Education level (patients)/ Career stage (doctors)	Highschool graduate or equivalent = 1<br>College sixth form graduate or equivalent = 3<br>University (bachelors) degree graduate or equivalent = 8<br>Postgraduate degree graduate = 2<br>Prefer not to say = 1	Consultant[3] = 8<br>Foundation Years doctor[4] = 4<br>Registrar[5] = 2
Geographic region	England/London: 5<br>England/East Midlands: 4<br>England/West Midlands: 1<br>England/North West: 1<br>England/South East: 1<br>England/Yorkshire & the Humber: 1<br>Wales/Cardiff: 1<br>Northern Ireland/Belfast: 1	England/East Midlands: 13<br>England/London: 1

Footnotes:

[1] Highschool graduates refer to students having completed GCSEs, typically aged 16

[2]College sixth form graduates refer to students having completed A-levels, typically aged 18.

[3]Consultant refers to a senior hospital doctor equivalent to an attending physician in the U.S. or a specialist doctor in other healthcare systems.

[4]Foundation year doctor refers to a junior doctor undergoing the first two years of post-medical school training.

[5]Registrar refers to a doctor undergoing specialist training.

Supplementary Table 2 Aggregate Demographic Data of Public Participants in the South African Co-Design Workshop

	**Public participants (n=22)**
Gender	Male = 7<br>Female = 15
Age	Mean = 40.64 years<br>SD = 11.24<br>Range = 20-61 years
Education level	Primary school graduate = 5[1]<br>Highschool graduate = 17[2]
Geographic region	All recruited from townships located within the Western Cape

Footnotes:

[1] Completed schooling up to Grade 7 (typically aged 13)

[2] Completed schooling up to the Grade 12 (typically aged 18).

e-Delphi

Supplementary Fig. 1. Overview of Delphi participants' nationalities.

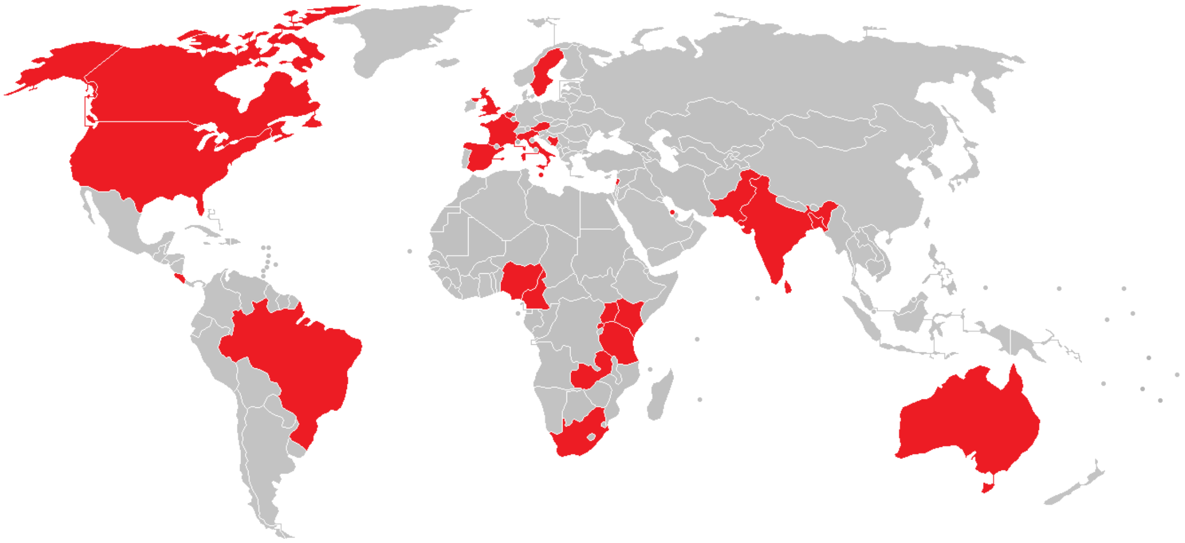

Countries shaded in red indicate the citizenship of at least one participant in the panel. Note that four participants reported dual citizenship.

Supplementary Table 3 List of native languages represented in the e-Delphi panel. Note that six participants reported more than one native language. 23 participants were non-native speakers of English.

Language	Participants with native fluency
Afrikaans	2
Arabic	3
Bantou	2
Bemba	1
Bengali	3
Bosnian	1
English	14
French	1
German	1
Hindi	3
Igbo	1
Italian	1
Kannada	2
Luganda	1
Maltese	1
Ngemba	1
Portughese	1
Sinhala	1
Spanish	3
Swahili	2
Swedish	1
Tamil	1
Urdu	1
Yoruba	3
Zulu	2

Supplementary Note 2: Metaphors that emerged from co-design workshops

Section 1: Diversity of microbial world

1. The microbial world is like a box of chocolates. Some chocolates are nice, some nasty - just like the different types of microbes.
2. The microbial world is like a range of mushrooms - some tasty, others dangerous. Similarly, some microbes are beneficial, while others are dangerous.
3. The microbial word is like an invisible rain forest with lots of diverse flora and fauna – invisible to the naked eye and with beautiful diversity.
4. The microbial world is as diverse as the many types of fish in the sea. Some are lovely Nemos, other killer sharks. Similarly, some microbes are beneficial, while others are dangerous.
5. The microbial world is like a garden that contains lots of different plants. Some plants like aggressive weeds destroy the ecosystem. Similarly, there are lots of different microbes, some of them are harmful and cause disease.
6. The microbial world is like a microscopic orchestra with lots of different instruments, all of which play their part. Similarly, there are lots of different types of tiny microbes, all of which have essential roles.
7. The microbial world is like chess board, where different pieces move in different ways. Together, they make up a whole team working together. Similarly, different types of microbes work in unison and are essential to the ecosystem.
8. The microbial world is like a kaleidoscope of hidden life – beautiful and diverse.
9. Microbes are like players in a video game – some are your friends, others are dangerous enemies.
10. Microbes are like an army of microscopic warriors, all with different strengths and weaknesses.
11. Microbes are diverse and can have different roles to play in maintaining the machinery of the human body. The presence of good microbes oils the body's engine and stops it going wrong. Bad microbes can put a spanner in the works.
12. There are lots of different microbes which, like humans, have different identities and speak different languages. When treating infections, we need to speak the language of specific microbes in order to target them in the right way.
13. The microbial world is like a complex employment setting. When at work, everyone has their own job role. Every person works to different rules or functions to make sure the overall company works. Similarly, all microbes have different roles to play.
14. Bacteria are like a football team - players all have different roles, strengths and weakness, but all are essential to the bigger team.

Section 2: Mechanisms of antibiotic resistance

15. Each use of antibiotics puts a brick in the wall to create a barrier that prevents us from reaching bacteria in the future.
16. Resistance is like constructing a flood barrier when it rains. Resistance is like the barrier that protects bacteria from antibiotic treatment.
17. Resistance is like a locked door that cannot be penetrated with our current antibiotics.

18. Having ineffective or partially effective antibiotics is like working with a blunt knife – ineffective and difficult.
19. Resistant bacteria are like hackers breaking through security software despite the programmers trying to stop them. Resistance is the skill that enables them to escape.
20. Resistant bacteria are like criminals wearing bulletproof vests for protection. The vests are like resistance, protecting bacteria from being caught or eliminated.
21. Antibiotic resistance is like a scientific or industrial revolution – just like machines and technologies have been upscaled over time, so have bacteria.
22. Drug-resistant bacteria are like artificial intelligence systems that have learned to outsmart their opponents. Just like AI systems have been upscaled over time, so have bacteria.
23. Bacteria learn to circle the wagons (have learned to defend themselves) and unite against our antibiotic weapons.
24. Just like weeds become resistant to weed killers so do bacteria become resistant to antibiotics.
25. Bacteria developing resistance is like people putting on a thick winter coat to avoid getting cold. Just like a coat protects from the cold, resistance protects bacteria from being killed.
26. Bacteria developing resistance is like people applying sun cream to avoid getting burned. Just like sun cream protects people from the sun, resistance protects bacteria from being killed.
27. Resistant bacteria are like players from opposing teams, who have learned your strategies and built stronger defences over time, thereby becoming unbeatable.
28. Antibiotic resistant bacteria are like soldiers, who have learned the enemy's tactics and developed new shields and defence mechanisms against their strategies and weapons.
29. Resistant bacteria are like war enemies that have developed armour, which cannot be penetrated by our existing antibiotics.
30. Drug resistance is like building a fortress to retreat to when the enemy threatens (e.g., to stop arrows). Resistance is like the fortress that protects bacteria from antibiotic treatment.
31. Resistant bacteria have developed hiding places that save them from our antibiotic attacks. Resistance is like the hideout that protects bacteria from antibiotic treatment.
32. Resistant bacteria are like weightlifters, who have developed new levels of strength to resist ordinary drugs.
33. Resistant bacteria are like students, who have passed their year and moved up to the next year or level.

Section 3: Antiviral efficacy

34. Using antibiotics to treat viral infections is like using the wrong key to unlock a door – it won't work and you might damage the lock.
35. Using antibiotics to treat viral infections is like using toilet cleaner on a carpet stain – it won't work and you the risk of ruining the carpet.
36. Using antibiotics to treat viral infections is like using water to clean paint brushes that were dipped in oil paints – futile.

37. Using antibiotics to treat viral infections is like using water on grease stains – a pointless effort and a waste of time.
38. Using antibiotics to treat viral infections is like using a pencil to write an email – inappropriate and potentially harmful.
39. Using antibiotics to treat viral infections is like using a leaky bucket to carry water – the water will pass through and you are left with nothing. It's a futile waste of resources.
40. Using antibiotics to treat viral infections is like eating soup with a fork – using the wrong tool is pointless.
41. Using antibiotics to treat viral infections is like a woman using a man's urinal – pointless and ridiculous.
42. Using antibiotics to treat viral infections is like placing an electric kettle on a fire – inappropriate and harmful.
43. Using antibiotics to treat viral infections is like watering a plastic plant – it superficially looks like the right thing to do but is ultimately pointless.
44. Using antibiotics to treat viruses is like giving chocolate to a person with lactose intolerance – it seems like a nice idea but ultimately makes them sick.
45. Using antibiotics to treat viruses is like using your tooth brush to clean your ears – inappropriate.
46. Using antibiotics to treat viral infections is like connecting the wrong pieces in a game of dominoes – they just don't work together.
47. Some of your weapons only work against a particular type of opponent in video games – just as antibiotics only work against bacterial infections.
48. Using antibiotics to treat viral infections is like using a bandage to treat a stomach ache – it seems like a good idea, but doesn't get to the source of the problem and is ultimately futile.
49. Using antibiotics to treat viral infections is like using aftershave on sunburned skin – using an inappropriate product only leads to harm.
50. Using antibiotics against viral infections is like putting diesel in a petrol (gas-powered) car – futile, potentially harmful and wasteful of resources.
51. Just like garden weeds require the right type of herbicide, we need to find the right type of medicine to treat infections.
52. Antibiotics cannot treat viral infections, because antibiotics don't speak the same language as viruses. Antibiotics only speak the language of bacteria. There might be different dialects for different types of bacteria.
53. Using antibiotics to treat viral infections is like calling someone by the wrong name and being surprised that they don't react – it's the wrong approach, which is ultimately futile.
54. Antibiotics cannot see viruses (either because viruses are too small or because they've found hiding places or are wearing cloaks of invisibility) – this means they don't work against viral infections.
55. We need to find the right way/direction to treat infections. Antibiotics don't know the way to reach viruses.
56. Using antibiotics against viruses is like drinking salt water when you're thirsty – using the wrong solution only does more harm than good.

Section 4: Body resistance

57. It's not the body that becomes resistant but the bacteria. Similarly, in the context of gardening, it's not the garden that becomes resistant to weed killers/herbicides, it's the individual weeds. Weeds can spread from one garden to another. This means that resistance can be found in gardens, which have never been treated with weed killers.
58. Bacteria are like little visitors living in your body. It's those visitors that become resistant, not your body. Visitors can move on or even multiply.

Section 5: Sharing of leftover antibiotics

59. Sharing leftover antibiotics is like sharing your toothbrush – it's inappropriate to share a personal or intimate item.
60. Sharing leftover antibiotics is like sharing your razor – it's inappropriate to share a personal or intimate item.
61. Sharing leftover antibiotics is like sharing your underwear – it's inappropriate to share a personal or intimate item, which was selected with your personal profile in mind.
62. Sharing leftover antibiotics is like using items past their expiry date. They had a clear purpose and should not be used beyond this purpose.

Section 6: Incomplete antibiotic course

63. Stopping antibiotics prematurely is like firefighters leaving before the fire is fully extinguished. The flames might seem gone, but embers remain, ready to reignite stronger. Similarly, not completing a treatment course as prescribed may mean that partially resistant bacteria remain and multiply.
64. Stopping antibiotics prematurely is like dropping out of a marathon half way through the race. Similarly, it is essential to complete the full course for optimal effectiveness.
65. Stopping antibiotics prematurely is like incomplete weeding of a garden – if you only pull out the visible weeds but leave the roots, weeds will grow back even more resilient. Similarly, stopping antibiotics too soon enables bacteria to return stronger.
66. Stopping antibiotics prematurely is like incomplete cancer treatment, which only leaves the most aggressive cancer cells, meaning that tumours will re-grow even stronger than the first.
67. Stopping antibiotics prematurely is like beating yourself up with a rock – unnecessary self-sabotage.
68. Stopping antibiotics prematurely is like digging your own grave. Stopping antibiotics too soon enables bacteria to return stronger, with grave consequences.
69. Stopping antibiotics prematurely is like sowing grass but then forgetting to water it. It's a half-measure that ultimately will be unsuccessful.
70. Stopping antibiotics prematurely is like residing in a house with no roof, leaving oneself vulnerable and exposed to harm.

Section 7: Following medical advice

71. Not following doctors' advice is like driving in an unknown area of town without your satnav on (or without following a map) – you will get lost and may end up in a ditch. Similarly, you need expert guidance to navigate complex treatment decisions.
72. Not following doctors' advice is like trying to bake a cake without a recipe – you might have a vague idea, but the result is unlikely to be optimal.

73. Not following doctors' advice is like going against traffic regulations - ill-advised, illegal and dangerous.

Section 8: Antibiotic use for minor infections

74. Using antibiotics for minor or self-limiting infections is like using a sledgehammer to kill a small insect – a disproportionate effort, which might do damage to surrounding areas.
75. Using antibiotics for minor or self-limiting infections is like shaving your head with a machete – disproportionate and potentially very dangerous.
76. Using antibiotics for minor or self-limiting infections is like using a large hammer to crack a small nut. A disproportionate effort, which might do more damage than good.
77. Using antibiotics for minor or self-limiting infections is similar to popping pills like candy. You might get sick from overuse.
78. Using antibiotics for minor infections is like wearing a full wet suit for going swimming at your local swimming pool – over the top and silly.
79. Using antibiotics for minor infections is like diving into a shallow bathtub – a disproportionate action that risks injury.
80. Using antibiotics for minor or self-limiting infections is like using a parachute every time you go down a flight of stairs – ridiculous and unnecessary.
81. Using antibiotics for self-limiting infections is like driving by car to visit your next-door neighbour – disproportionate, unnecessary and wasteful of resources.
82. Infections are like fires that must be contained. Some fires will burn out by themselves. We must protect our fire extinguishers (antibiotics) and only use them for emergencies (dangerous infections). For example, it would be disproportionate and dangerous to use a fire extinguisher to blow out a candle.
83. In video games, you only have a limited number of weapons available, so you need to save them for the real enemy – just like you need to save antibiotics for the most serious infections.
84. Working antibiotics are like goalkeepers that prevent the opposite team from scoring. Goalies shouldn't get involved in every tackle of the game. We need to save our goalies for when the opposite team breaks through our final defences.
85. We need to save our precious resource of antibiotics for when they are really needed just like we need to keep our powder dry in war.

Section 9: Infection prevention

86. It's best to avoid infections (opponents in video games) altogether – you could do this by using safer paths in the game. Vaccination and infection prevention could be such safer paths.
87. We need more life boats (antibiotics), but if possible we should avoid hitting the iceberg (getting infected) in the first place.
88. We should build damns (vaccinate, prevent infection) rather than use our (antibiotic) lifeboats.
89. We need the roadside assistance and breakdown recovery service, but it's better to maintain the car and avoid a call-out. Similarly, we should reserve antibiotics for emergencies.

Supplementary Note 3: Metaphors rated across the three e-Delphi rounds

Original metaphor ratings

Supplementary Table 4 Overview of item ratings across rounds for original metaphors that were generated during Co-design workshops

M = Median, D = Disagreement (yes/no), Rewording = indicates whether an item was re-worded

		Round 1			Round 2			Round 3			Outcome
**Item**	**Original item text**	**M**	**D**	**Rewording**	**M**	**D**	**Rewording**	**M**	**D**	**Rewording**	
**Section 1: Diversity of microbial world**											
1.a	The microbial world is like a box of chocolates. Some chocolates are nice, some nasty - just like the different types of microbes.	4	No	The world of germs is a box of chocolates - some nice, others nasty.	3	No	N/A	N/A	N/A	N/A	Inappropriate
1.b	The microbial world is like a range of mushrooms - some tasty, others dangerous. Similarly, some microbes are beneficial, while others are dangerous.	6	No	Some germs are good, others dangerous—just like mushrooms	6.5	No	N/A	N/A	N/A	N/A	Unsure
1.c	The microbial word is like an invisible rain forest with lots of diverse flora and fauna – invisible to the naked eye and with beautiful diversity.	5	No	The world of germs is a hidden jungle of life, filled with both predators and protectors.	5.5	No	N/A	N/A	N/A	N/A	Unsure
1.d	The microbial world is as diverse as the many types of fish in the sea. Some are lovely Nemos, other killer sharks. Similarly, some microbes are beneficial, while others are dangerous.	6	No	The world of germs is as diverse as the many types of fish in the sea. Some are lovely Nemos, other killer sharks.	6	No	N/A	N/A	N/A	N/A	Unsure

1.e	The microbial world is like a garden that contains lots of different plants. Some plants like aggressive weeds destroy the ecosystem. Similarly, there are lots of different microbes, some of them are harmful and cause disease.	6	No	The world of germs is a garden with both flowers and weeds.	6	No	N/A	N/A	N/A	N/A	Unsure
1.f	The microbial world is like a microscopic orchestra with lots of different instruments, all of which play their part. Similarly, there are lots of different types of tiny microbes, all of which have essential roles.	4	No	The world of germs forms a microscopic orchestra—each plays a role.	3	No	N/A	N/A	N/A	N/A	Inappropriate
1.g	The microbial world is like chess board, where different pieces move in different ways. Together, they make up a whole team working together. Similarly, different types of microbes work in unison and are essential to the ecosystem.	4	No	The world of germs is a chess board, with different pieces working as a team.	3	No	N/A	N/A	N/A	N/A	Inappropriate
1.h	The microbial world is like a kaleidoscope of hidden life – beautiful and diverse.	4	No	The world of germs is a living kaleidoscope.	3	No	N/A	N/A	N/A	N/A	Inappropriate
1.i	Microbes are like players in a video game – some are your friends, others are dangerous enemies.	6	No	Germs are video game characters—some allies, some foes.	5.5	Yes	N/A	6	Yes	N/A	Unsure
1.j	Microbes are like an army of microscopic warriors, all with different strengths and weaknesses.	4	No	Germs are microscopic warriors with different strengths and weaknesses.	3	No	N/A	N/A	N/A	N/A	Unsure

1.k	Microbes are diverse and can have different roles to play in maintaining the machinery of the human body. The presence of good microbes oils the body's engine and stops it going wrong. Bad microbes can put a spanner in the works.	6	No	Germs keep the body’s engine running—or can break it down.	6	No	N/A	N/A	N/A	N/A	Unsure
1.l	There are lots of different microbes which, like humans, have different identities and speak different languages. When treating infections, we need to speak the language of specific microbes in order to target them in the right way.	6	No	Germs have different identities and speak many different languages	6	No	N/A	N/A	N/A	N/A	Inappropriate
1.m	The microbial world is like a complex employment setting. When at work, everyone has their own job role. Every person works to different rules or functions to make sure the overall company works. Similarly, all microbes have different roles to play.	4	No	The world of germs is a workplace—each germ has a job.	4	No	N/A	N/A	N/A	N/A	Unsure
1.n	Bacteria are like a football team - players all have different roles, strengths and weakness, but all are essential to the bigger team.	5	No	Germs are a football team—different roles, one goal.	4	No	N/A	N/A	N/A	N/A	Unsure
**Section 2: Mechanisms of antibiotic resistance**											
2.a	Each use of antibiotics puts a brick in the wall to create a barrier that prevents us from reaching bacteria in the future.	3	No	N/A	N/A	N/A	N/A	N/A	N/A	N/A	Inappropriate

2.b	Resistance is like constructing a flood barrier when it rains. Resistance is like the barrier that protects bacteria from antibiotic treatment.	4	No	Antibiotic resistance is a flood barrier blocking treatment.	3	No	N/A	N/A	N/A	N/A	Inappropriate
2.c	Resistance is like a locked door that cannot be penetrated with our current antibiotics.	5	No	Antibiotic resistance is a locked door that cannot be penetrated with our current antibiotics.	4.5	No	N/A	N/A	N/A	N/A	Unsure
2.d	Having ineffective or partially effective antibiotics is like working with a blunt knife – ineffective and difficult.	6	No	Ineffective antibiotics are blunt tools.	6	No	N/A	N/A	N/A	N/A	Unsure
2.e	Resistant bacteria are like hackers breaking through security software despite the programmers trying to stop them. Resistance is the skill that enables them to escape.	5	No	Antibiotic-resistant bacteria are hackers breaking through security.	5	No	N/A	N/A	N/A	N/A	Unsure
2.f	Resistant bacteria are like criminals wearing bulletproof vests for protection. The vests are like resistance, protecting bacteria from being caught or eliminated.	6	No	Antibiotic resistance is a bulletproof vest for bacteria.	6	No	N/A	N/A	N/A	N/A	Unsure
2.g	Antibiotic resistance is like a scientific or industrial revolution – just like machines and technologies have been upscaled over time, so have bacteria.	4	No	Antibiotic resistance is an industrial revolution where bacteria level up.	3	No	N/A	N/A	N/A	N/A	Inappropriate
2.h	Drug-resistant bacteria are like artificial intelligence systems that have learned to outsmart their opponents. Just like AI systems have been upscaled over time, so have bacteria.	4	No	Antibiotic-resistant bacteria are Artificial Intelligence that has outsmarted us.	4	No	N/A	N/A	N/A	N/A	Unsure

2.i	Bacteria learn to circle the wagons (have learned to defend themselves) and unite against our antibiotic weapons.	4	No	Bacteria learn to circle the wagons and unite against our antibiotic weapons.	3	No	N/A	N/A	N/A	N/A	Inappropriate
2.j	Just like weeds become resistant to weed killers so do bacteria become resistant to antibiotics.	7	No	Weeds resist weed killer—bacteria resist antibiotics.	7	No	N/A	N/A	N/A	N/A	**Appropriate**
2.k	Bacteria developing resistance is like people putting on a thick winter coat to avoid getting cold. Just like a coat protects from the cold, resistance protects bacteria from being killed.	6	No	Antibiotic resistance is a thick protective coat for bacteria that blocks antibiotics.	6	No	N/A	N/A	N/A	N/A	Unsure
2.l	Bacteria developing resistance is like people applying sun cream to avoid getting burned. Just like sun cream protects people from the sun, resistance protects bacteria from being killed.	6	No	Antibiotic resistance is protective sunblock for bacteria	6	No	N/A	N/A	N/A	N/A	Unsure
2.m	Resistant bacteria are like players from opposing teams, who have learned your strategies and built stronger defences over time, thereby becoming unbeatable.	6	No	Antibiotic-resistant bacteria are opponents, who have learned your strategies and become unbeatable.	6	No	N/A	N/A	N/A	N/A	Unsure
2.n	Antibiotic resistant bacteria are like soldiers, who have learned the enemy's tactics and developed new shields and defence mechanisms against their strategies and weapons.	6	No	This item was combined with 2.o due to overlapping contents	N/A	N/A	N/A	N/A	N/A	N/A	Inappropriate
2.o	Resistant bacteria are like war enemies that have developed armour, which cannot be penetrated by our existing antibiotics.	6	No	Antibiotic-resistant bacteria are war enemies with impenetrable armour.	6	No	Antibiotic-resistant bacteria are intruders wearing smart armour that upgrades itself when exposed to medicine.	7	No	Antibiotic-resistant bacteria are intruders wearing smart armour that upgrades itself	**Appropriate**

										when exposed to antibiotics.	
2.p	Drug resistance is like building a fortress to retreat to when the enemy threatens (e.g., to stop arrows). Resistance is like the fortress that protects bacteria from antibiotic treatment.	5	No	Antibiotic resistance is an impenetrable fortress that protects bacteria from antibiotic treatment.	4	No	N/A	N/A	N/A	N/A	Unsure
2.q	Resistant bacteria have developed hiding places that save them from our antibiotic attacks. Resistance is like the hideout that protects bacteria from antibiotic treatment.	5	No	Antibiotic resistance is a hideout for bacteria to avoid antibiotics.	3	No	N/A	N/A	N/A	N/A	Inappropriate
2.r	Resistant bacteria are like weightlifters, who have developed new levels of strength to resist ordinary drugs.	5	No	Antibiotic-resistant bacteria are weightlifters, who have developed new levels of strength to resist ordinary drugs.	5	No	N/A	N/A	N/A	N/A	Unsure
2.s	Resistant bacteria are like students, who have passed their year and moved up to the next year or level.	3	No	N/A	N/A	N/A	N/A	N/A	N/A	N/A	Inappropriate
**Section 3: Antiviral efficacy**											
3.a	Using antibiotics to treat viral infections is like using the wrong key to unlock a door – it won't work and you might damage the lock.	7	No	Using antibiotics for viruses is using the wrong key to unlock a door.	8	No	N/A	N/A	N/A	N/A	**Appropriate**
3.b	Using antibiotics to treat viral infections is like using toilet cleaner on a carpet stain – it won't work and you the risk of ruining the carpet.	5	No	Using antibiotics for viruses is like using toilet cleaner on carpet.	4	No	N/A	N/A	N/A	N/A	Unsure

3.c	Using antibiotics to treat viral infections is like using water to clean paint brushes that were dipped in oil paints – futile.	5	No	Using antibiotics for viruses is like rinsing oil paint with water.	4	No	N/A	N/A	N/A	N/A	Unsure
3.d	Using antibiotics to treat viral infections is like using water on grease stains – a pointless effort and a waste of time.	5	No	Using antibiotics on viruses is like water on grease.	5	No	N/A	N/A	N/A	N/A	Unsure
3.e	Using antibiotics to treat viral infections is like using a pencil to write an email – inappropriate and potentially harmful.	4	No	Using antibiotics for viruses is writing an email with a pencil.	3	No	N/A	N/A	N/A	N/A	Inappropriate
3.f	Using antibiotics to treat viral infections is like using a leaky bucket to carry water – the water will pass through and you are left with nothing. It's a futile waste of resources.	5	Yes	Using antibiotics for viruses is using a leaky bucket.	3	No	N/A	N/A	N/A	N/A	Inappropriate
3.g	Using antibiotics to treat viral infections is like eating soup with a fork – using the wrong tool is pointless.	6	No	Using antibiotics for viruses is eating soup with a fork.	6	No	N/A	N/A	N/A	N/A	Unsure
3.h	Using antibiotics to treat viral infections is like a woman using a man's urinal – pointless and ridiculous.	2	No	N/A	N/A	N/A	N/A	N/A	N/A	N/A	Inappropriate
3.i	Using antibiotics to treat viral infections is like placing an electric kettle on a fire – inappropriate and harmful.	4	No	Using antibiotics for viruses is placing an electric kettle on a fire to heat.	3.5	No	N/A	N/A	N/A	N/A	Inappropriate
3.j	Using antibiotics to treat viral infections is like watering a plastic plant – it superficially looks like the right thing to do but is ultimately pointless.	6	No	Using antibiotics for viruses is watering fake plants.	5	No	N/A	N/A	N/A	N/A	Unsure

3.k	Using antibiotics to treat viruses is like giving chocolate to a person with lactose intolerance – it seems like a nice idea but ultimately makes them sick.	3	No	N/A	N/A	N/A	N/A	N/A	N/A	N/A	Inappropriate
3.l	Using antibiotics to treat viruses is like using your tooth brush to clean your ears –inappropriate.	4	No	Using antibiotics for viruses is brushing ears with a toothbrush.	3.5	No	N/A	N/A	N/A	N/A	Inappropriate
3.m	Using antibiotics to treat viral infections is like connecting the wrong pieces in a game of dominoes – they just don't work together.	3	No	N/A	N/A	N/A	N/A	N/A	N/A	N/A	Inappropriate
3.n	Some of your weapons only work against a particular type of opponent in video games – just as antibiotics only work against bacterial infections.	5	No	Using antibiotics for viruses is using the wrong type of weapon against a video game opponent.	4	No	N/A	N/A	N/A	N/A	Unsure
3.o	Using antibiotics to treat viral infections is like using a bandage to treat a stomach ache – it seems like a good idea, but doesn't get to the source of the problem and is ultimately futile.	5	No	Using antibiotics for viruses is like using a bandage for a stomach ache	5	No	N/A	N/A	N/A	N/A	Unsure
3.p	Using antibiotics to treat viral infections is like using aftershave on sunburned skin – using an inappropriate product only leads to harm.	5	No	Using antibiotics for viruses is like using aftershave on sunburn.	3.5	No	N/A	N/A	N/A	N/A	Inappropriate
3.q	Using antibiotics against viral infections is like putting diesel in a petrol (gas-powered) car – futile, potentially harmful and wasteful of resources.	7	No	Using antibiotics for viruses is like putting diesel in a petrol car.	7	No	N/A	N/A	N/A	N/A	**Appropriate**

3.r	Just like garden weeds require the right type of herbicide, we need to find the right type of medicine to treat infections.	5	No	Using antibiotics for viruses is using the wrong weed killer on garden pests.	5	No	N/A	N/A	N/A	N/A	Unsure
3.s	Antibiotics cannot treat viral infections, because antibiotics don't speak the same language as viruses. Antibiotics only speak the language of bacteria. There might be different dialects for different types of bacteria.	5	No	Using antibiotics for viruses is speaking the wrong language.	5	No	Using antibiotics for viruses is like shouting in the wrong language at a locked door—it won't open, and you might just make things worse.	4	Yes	N/A	Unsure
3.t	Using antibiotics to treat viral infections is like calling someone by the wrong name and being surprised that they don't react – it's the wrong approach, which is ultimately futile.	6	No	Using antibiotics for viruses calling someone the wrong name.	4.5	No	N/A	N/A	N/A	N/A	Unsure
3.u	Antibiotics cannot see viruses (either because viruses are too small or because they've found hiding places or are wearing cloaks of invisibility) – this means they don't work against viral infections.	4	No	Using antibiotics for viruses is sending a guard after an invisible enemy.	4	No	N/A	N/A	N/A	N/A	Unsure
3.v	We need to find the right way/direction to treat infections. Antibiotics don't know the way to reach viruses.	4	No	Using antibiotics for viruses is like sending your guard on the wrong path after an enemy.	4	No	N/A	N/A	N/A	N/A	Unsure
3.w	Using antibiotics against viruses is like drinking salt water when you're thirsty – using the wrong solution only does more harm than good.	6	No	Using antibiotics for viruses is drinking salt water when thirsty.	5	No	N/A	N/A	N/A	N/A	Unsure
**Section 4: Body resistance**											

4.a	It’s not the body that becomes resistant but the bacteria. Similarly, in the context of gardening, it’s not the garden that becomes resistant to weed killers/herbicides, it’s the individual weeds. Weeds can spread from one garden to another. This means that resistance can be found in gardens, which have never been treated with weed killers.	6	No	It’s not the body that becomes resistant but the bacteria. Similarly, it’s not the garden that becomes resistant to weed killers, it’s the individual weeds.	6	No	N/A	N/A	N/A	N/A	Unsure
4.b	Bacteria are like little visitors living in your body. It’s those visitors that become resistant, not your body. Visitors can move on or even multiply.	6	No	Bacteria are little visitors in your body- they can change (become resistant), move on, or multiply.	6	No	N/A	N/A	N/A	N/A	Unsure
**Section 5: Sharing of leftover antibiotics**											
5.a	Sharing leftover antibiotics is like sharing your toothbrush – it’s inappropriate to share a personal or intimate item.	5	No	Sharing leftover antibiotics is like sharing a toothbrush - inappropriate.	5	No	N/A	N/A	N/A	N/A	Unsure
5.b	Sharing leftover antibiotics is like sharing your razor – it’s inappropriate to share a personal or intimate item.	4	No	Sharing leftover antibiotics is like sharing your razor - inappropriate.	4	No	N/A	N/A	N/A	N/A	Unsure
5.c	Sharing leftover antibiotics is like sharing your underwear – it’s inappropriate to share a personal or intimate item, which was selected with your personal profile in mind.	5	No	Sharing leftover antibiotics is like sharing your underwear - inappropriate.	4	No	N/A	N/A	N/A	N/A	Unsure
5.d	Sharing leftover antibiotics is like using items past their expiry date. They had a clear purpose and should not be used beyond this purpose.	4	No	Sharing leftover antibiotics is like using expired products - wrong and risky.	4	No	N/A	N/A	N/A	N/A	Unsure
**Section 6: Self-directed premature termination of treatment course**											

6.a	Stopping antibiotics prematurely is like firefighters leaving before the fire is fully extinguished. The flames might seem gone, but embers remain, ready to reignite stronger. Similarly, not completing a treatment course as prescribed may mean that partially resistant bacteria remain and multiply.	7	No	An unfinished course of antibiotics is a fire half-out. Embers remain, ready to flare up again even stronger	8	No	An unfinished course of antibiotics is a fire half-out. Sparks remain, ready to flare up again even stronger.	N/A	N/A	N/A	**Appropriate**
6.b	Stopping antibiotics prematurely is like dropping out of a marathon half way through the race. Similarly, it is essential to complete the full course for optimal effectiveness.	5	No	An unfinished course of antibiotics is a half-completed race.	4	No	N/A	N/A	N/A	N/A	Unsure
6.c	Stopping antibiotics prematurely is like incomplete weeding of a garden – if you only pull out the visible weeds but leave the roots, weeds will grow back even more resilient. Similarly, stopping antibiotics too soon enables bacteria to return stronger.	7	No	An unfinished course of antibiotics is a partially weeded garden with roots left behind that will grow back even more resilient.	7	No	N/A	N/A	N/A	N/A	**Appropriate**
6.d	Stopping antibiotics prematurely is like incomplete cancer treatment, which only leaves the most aggressive cancer cells, meaning that tumours will re-grow even stronger than the first.	5	No	An unfinished course of antibiotics is like incomplete cancer treatment that leaves the most aggressive cells to grow and multiply.	6	No	N/A	N/A	N/A	N/A	Unsure
6.e	Stopping antibiotics prematurely is like beating yourself up with a rock – unnecessary self-sabotage.	3	No	N/A	N/A	N/A	N/A	N/A	N/A	N/A	Inappropriate

6.f	Stopping antibiotics prematurely is like digging your own grave. Stopping antibiotics too soon enables bacteria to return stronger, with grave consequences.	5	No	An unfinished course of antibiotics is a grave you're digging for yourself.	4	No	N/A	N/A	N/A	N/A	Unsure
6.g	Stopping antibiotics prematurely is like sowing grass but then forgetting to water it. It's a half-measure that ultimately will be unsuccessful.	4	No	An unfinished course of antibiotics is sowing grass without watering it.	3	No	N/A	N/A	N/A	N/A	Inappropriate
6.h	Stopping antibiotics prematurely is like residing in a house with no roof, leaving oneself vulnerable and exposed to harm.	3	No	N/A	N/A	N/A	N/A	N/A	N/A	N/A	Inappropriate
**Section 7: Following medical advice**											
7.a	Not following doctors' advice is like driving in an unknown area of town without your satnav on (or without following a map) – you will get lost and may end up in a ditch. Similarly, you need expert guidance to navigate complex treatment decisions.	6	No	Ignoring doctors' advice is throwing out a friend's map in unfamiliar territory.	6	No	N/A	N/A	N/A	N/A	Unsure
7.b	Not following doctors' advice is like trying to bake a cake without a recipe – you might have a vague idea, but the result is unlikely to be optimal.	7	No	Ignoring doctors' advice is baking without a recipe.	7	No	N/A	N/A	N/A	N/A	**Appropriate**
7.c	Not following doctors' advice is like going against traffic regulations - ill-advised, illegal and dangerous.	6	Yes	Ignoring doctors' advice is like breaking traffic laws.	5	No	Ignoring medical advice is like driving through a red light at a busy crossing - you might survive, but you're putting others in danger.	6	Yes	N/A	Unsure
**Section 8: Antibiotic use for minor infections**											

8.a	Using antibiotics for minor or self-limiting infections is like using a sledgehammer to kill a small insect – a disproportionate effort, which might do damage to surrounding areas.	7	No	Using antibiotics for minor infections is using a sledgehammer to kill a fly.	7	No	N/A	N/A	N/A	N/A	**Appropriate**
8.b	Using antibiotics for minor or self-limiting infections is like shaving your head with a machete – disproportionate and potentially very dangerous.	6	No	Using antibiotics for minor infections is like using a machete to shave.	6	No	N/A	N/A	N/A	N/A	Unsure
8.c	Using antibiotics for minor or self-limiting infections is like using a large hammer to crack a small nut. A disproportionate effort, which might do more damage than good.	7	No	Using antibiotics for minor infections is using a hammer to crack a peanut.	7	No	N/A	N/A	N/A	N/A	**Appropriate**
8.d	Using antibiotics for minor or self-limiting infections is similar to popping pills like candy. You might get sick from overuse.	4	No	Using antibiotics for minor infections is popping pills like candy.	4	No	N/A	N/A	N/A	N/A	Unsure
8.e	Using antibiotics for minor infections is like wearing a full wet suit for going swimming at your local swimming pool – over the top and silly.	4	No	Using antibiotics for minor infections is wearing a wet suit to the pool.	3.5	No	N/A	N/A	N/A	N/A	Inappropriate
8.f	Using antibiotics for minor infections is like diving into a shallow bathtub – a disproportionate action that risks injury.	5	No	Using antibiotics for minor infections is a dive into a shallow bathtub.	5	No	N/A	N/A	N/A	N/A	Unsure
8.g	Using antibiotics for minor or self-limiting infections is like using a parachute every time you go down a flight of stairs – ridiculous and unnecessary.	5	No	Using antibiotics for minor infections is like using a parachute to go down a flight of stairs.	5.5	No	N/A	N/A	N/A	N/A	Unsure

8.h	Using antibiotics for self-limiting infections is like driving by car to visit your next-door neighbour – disproportionate, unnecessary and wasteful of resources.	6	No	Using antibiotics for minor infections is like driving next door.	5.5	No	N/A	N/A	N/A	N/A	Unsure
8.i	Infections are like fires that must be contained. Some fires will burn out by themselves. We must protect our fire extinguishers (antibiotics) and only use them for emergencies (dangerous infections). For example, it would be disproportionate and dangerous to use a fire extinguisher to blow out a candle.	7	No	Using antibiotics for minor infections is using a fire extinguisher to blow out a candle.	7.5	No	N/A	N/A	N/A	N/A	**Appropriate**
8.j	In video games, you only have a limited number of weapons available, so you need to save them for the real enemy – just like you need to save antibiotics for the most serious infections.	6	No	Using antibiotics for minor infection is wasting weapons in a video game.	6	No	N/A	N/A	N/A	N/A	Unsure
8.k	Working antibiotics are like goalkeepers that prevent the opposite team from scoring. Goalies shouldn't get involved in every tackle of the game. We need to save our goalies for when the opposite team breaks through our final defences.	6	No	Using antibiotics for minor infection is using goalkeepers in every tackle.	6	No	N/A	N/A	N/A	N/A	Unsure
8.l	We need to save our precious resource of antibiotics for when they are really needed just like we need to keep our powder dry in war.	4	No	Using antibiotics for minor infection is wasting gun power. We need to keep our powder dry for the big wars.	3	No	N/A	N/A	N/A	N/A	Inappropriate
**Section 9: Infection prevention**											

9.a	It's best to avoid infections (opponents in video games) altogether – you could do this by using safer paths in the game. Vaccination and infection prevention could be such safer paths.	5	No	We can save antibiotics by avoiding infection just like we avoid dangerous opponents in video games.	5	No	N/A	N/A	N/A	N/A	Unsure
9.b	We need more life boats (antibiotics), but if possible we should avoid hitting the iceberg (getting infected) in the first place.	6	Yes	We can save our antibiotic life jackets by avoiding a boat crash and getting infected in the first place.	6	No	N/A	N/A	N/A	N/A	Unsure
9.c	We should build damns (vaccinate, prevent infection) rather than use our (antibiotic) lifeboats.	5	No	We can save our antibiotic lifeboats by building dams and avoiding infection.	4	No	N/A	N/A	N/A	N/A	Unsure
9.d	We need the roadside assistance and breakdown recovery service, but it's better to maintain the car and avoid a call-out. Similarly, we should reserve antibiotics for emergencies.	6	No	We can save our antibiotics by avoiding infection just like we avoid emergency services by maintaining our car.	7	No	N/A	N/A	N/A	N/A	**Appropriate**

New metaphor ratings

Supplementary Table 5 Overview of item ratings across rounds for metaphors that were newly suggested by e-Delphi participants in Round 1

		Round 2			Round 3			Outcome
**Item**	**Original item text**	**Median**	**Disagree**	**Rewording**	**Median**	**Disagree**	**Rewording**	
**Section 1: Diversity of microbial world**								
N1.1	The world of germs is a garden full of diverse insects - some helpful, a few dangerous.	7	No	N/A	N/A	N/A	N/A	**Appropriate**
N1.2	The world of germs is a classroom full of individuals with unique personalities, strengths, and quirks.	5	Yes	N/A	5	Yes	N/A	Unsure
N1.3	Communities of germs are like human neighbourhoods, made up of diverse residents including a few trouble-makers.	7	Yes	Communities of germs are like human neighbourhoods, made up of diverse residents including a few trouble-makers with the potential to cause chaos.	7	No	N/A	**Appropriate**
N1.4	The world of germs is a library filled with different books, each with its own story, role, and value.	6	Yes	N/A	6	Yes	N/A	Unsure
N1.5	Coexisting with germs is a dance—there is no rivalry, only living beings adjusting to each other in rhythm and balance.	4	Yes	N/A	3	No	N/A	Inappropriate
N1.6	The world of germs is a choir full of unique voices, from deep bass to high soprano.	3.5	No	N/A	N/A	N/A	N/A	Inappropriate
N1.7	Germs are like diverse smartphone apps—most run quietly in the background, others crash the system.	4.5	No	N/A	4	No	N/A	Unsure

N1.8	The world of germs is a tapestry, with invisible threads forming an intricate fabric of life.	3	No	N/A	N/A	N/A	N/A	Inappropriate
N1.9	The world of germs is a puzzle, where many different pieces form a complete picture.	4	Yes	N/A	4	Yes	N/A	Unsure
N1.10	Germs are like rivers feeding into a lake—each one contributing to the larger system.	3	No	N/A	N/A	N/A	N/A	Inappropriate
N1.11	The world of germs is a starry sky—countless and varied.	4	Yes	N/A	4	Yes	N/A	Unsure
N1.12	Germs are like cooking spices—most add flavours while some spoil the dish.	6	Yes	Germs are like spices—perfect in the right dish, but they can ruin the meal if they don't belong.	6	No	N/A	Unsure
N1.13	Germs are as varied as animals, living in balance but fighting back to survive if threatened.	6.5	Yes	Germs are as varied as wild animals, living in balance but adapting to survive if threatened.	7	No	N/A	**Appropriate**
**Section 2: Mechanisms of antibiotic resistance**								
N2.1	Antibiotic-resistant bacteria change the locks to effective treatment, making our antibiotic keys useless.	7	No	N/A	N/A	N/A	N/A	**Appropriate**
N2.2	Antibiotic-resistant bacteria are shapeshifters, changing form just enough to evade our defences.	6	Yes	Antibiotic-resistant bacteria are shapeshifters that change form when exposed to antibiotics - just enough to evade our defences.	6	Yes	N/A	Unsure
N2.3	Antibiotic-resistant bacteria are drought-resistant plants, growing in rough conditions.	3	No	N/A	N/A	N/A	N/A	Inappropriate
N2.4	Antibiotic resistance is like a game of dodgeball, where bacteria learn the movements of the ball and eventually dodge it entirely.	5	Yes	Antibiotic resistance is like a ball elimination game, where bacteria learn the movements of the ball and eventually dodge it entirely.	5	No	N/A	Unsure

N2.5	Antibiotic-resistant bacteria retain their strength like Samson without Delilah.	2	No	N/A	N/A	N/A	N/A	Inappropriate
N2.6	Antibiotic resistance is pressure and counter-pressure, where bacteria push back against antibiotic treatment.	5	Yes	N/A	5	Yes	N/A	Unsure
N2.7	Antibiotic resistance is fishing with a holey net, allowing resistant bacteria to slip through.	5	Yes	Antibiotic-resistant bacteria are like fish that have learned to slip through the holes in our nets.	5	Yes	N/A	Unsure
N2.8	Antibiotic resistance is like a clogged pipe—what once flowed smoothly is now blocked.	4	Yes	N/A	3	No	N/A	Inappropriate
N2.9	Antibiotic-resistant bacteria are a moving target for our antibiotic weapons.	3	Yes	N/A	2	No	N/A	Inappropriate
N2.10	Antibiotics are like shoes in a muddy field, wearing thin and losing effectiveness.	3	No	N/A	N/A	N/A	N/A	Inappropriate
N2.11	Antibiotic-resistant bacteria are rebellious teenagers who've outsmarted their parents.	2	No	N/A	N/A	N/A	N/A	Inappropriate
N2.12	Bacteria are like flowing water, finding creative ways around barriers.	7	No	N/A	N/A	N/A	N/A	**Appropriate**
N2.13	Bacteria are escape artists slipping through the cracks in our treatment.	5	Yes	N/A	5	Yes	N/A	Unsure
N2.14	Bacteria learn to pick the locks of our medicine cabinets. All antibiotic use trains them to break in faster.	3	No	N/A	N/A	N/A	N/A	Inappropriate
N2.15	Antibiotic resistance is global warming in medicine	3.5	Yes	N/A	3	No	N/A	Inappropriate
**Section 3: Antiviral efficacy**								

N3.1	Using antibiotics for viruses is like entering the wrong password for your username.	8	No	N/A	N/A	N/A	N/A	**Appropriate**
N3.2	Using antibiotics for viruses is using the wrong charger for your phone.	8	No	N/A	N/A	N/A	N/A	**Appropriate**
N3.3	Using antibiotics for viruses is putting earphones over your eyes.	6.5	Yes	N/A	7	No	N/A	**Appropriate**
N3.4	Using antibiotics for viruses is adding parsley to a dish that calls for coriander.	2	No	N/A	N/A	N/A	N/A	Inappropriate
N3.5	Using antibiotics for viruses is changing the oil in your car when you have a flat tyre.	7	No	N/A	N/A	N/A	N/A	**Appropriate**
N3.6	Using antibiotics for viruses is using salt to sweeten your coffee.	7	No	N/A	N/A	N/A	N/A	**Appropriate**
N3.7	Using antibiotics for viruses is like using a wrecking ball to dismantle a tent.	3.5	Yes	Using antibiotics for minor infections is like using a wrecking ball to dismantle a tent.	4	Yes	N/A	Unsure
N3.8	Using antibiotics for viruses is using an umbrella to protect from wind.	4.5	Yes	Using antibiotics for viruses is using an umbrella to protect from storm.	3	No	N/A	Inappropriate
N3.9	Using antibiotics for viruses is wearing a thick winter coat for sun protection.	4	Yes	N/A	3	No	N/A	Inappropriate
N3.10	Using antibiotics for viruses is cleaning your teeth with a hairbrush.	6	Yes	N/A	7	Yes	N/A	Appropriate *but not recommended
N3.11	Using antibiotics for viruses is like a plane ticket for someone without passport.	2	No	N/A	N/A	N/A	N/A	Inappropriate
**Section 4: Body resistance**								

N4.1	The body is a universe made of many worlds—each microbiome is its own ecosystem, inhabited by bacteria. These bacteria, not the body, adapt and evolve.	4.5	Yes	N/A	4	Yes	N/A	Unsure
N4.2	Bacteria are tenants in our bodies—some become resistant, refuse to move out or multiply and spread.	6	No	N/A	6	Yes	N/A	Unsure
N4.3	Bacteria are offenders using the human body as a hiding place to escape justice.	2	No	N/A	N/A	N/A	N/A	Inappropriate
N4.4	Bacteria are tourists in our bodies—some break the rules, outstay their welcome and cause trouble.	4.5	Yes	N/A	4	No	N/A	Unsure
N4.5	Bacteria are evolving species in a rainforest—the environment stays the same while bacteria adapt over time.	4	Yes	N/A	4	No	N/A	Unsure
N4.6	Just like a bad apple spoils the whole bushel, a few resistant bacteria can affect the wider population—changing the balance and spreading resistance to others.	7	No	N/A	N/A	N/A	N/A	**Appropriate**
N4.7	Like a single missing roof tile can leave a room drenched, one resistant bacterium can compromise the protection antibiotics offer.	7	No	N/A	N/A	N/A	N/A	**Appropriate**
**Section 5: Sharing of leftover antibiotics**								
N5.1	Sharing leftover antibiotics is like lending someone a personally tailored suit.	3	Yes	N/A	3	No	N/A	Inappropriate

N5.2	Sharing leftover antibiotics is using your house key for someone else’s door.	7	No	N/A	N/A	N/A	N/A	**Appropriate**
N5.3	Sharing leftover antibiotics is like giving someone your prescription glasses.	7	Yes	N/A	6	Yes	N/A	Unsure
N5.4	Sharing leftover antibiotics is like giving your friend sweets when what they really need is salad.	3.5	Yes	N/A	3	No	N/A	Inappropriate
N5.5	Sharing leftover antibiotics is like serving someone last week’s leftovers.	3	Yes	Sharing leftover antibiotics is like serving someone leftovers from two weeks ago.	4	No	N/A	Unsure
N5.6	Sharing leftover antibiotics is like sharing your PIN code for someone else’s phone.	6	Yes	N/A	6	Yes	N/A	Unsure
N5.7	Sharing leftover antibiotics is like sending your plumber when someone needs an electrician.	6	Yes	N/A	6	No	N/A	Unsure
N5.8	Sharing leftover antibiotics is like giving a child’s life jacket to an adult.	3	Yes	Sharing leftover antibiotics is like giving an adult-sized life jacket to a child.	3	No	N/A	Inappropriate
**Section 6: Self-directed premature termination of treatment course**								
N6.1	An unfinished course of antibiotics is a half-constructed house with no roof.	7	No	N/A	N/A	N/A	N/A	**Appropriate**
N6.2	An unfinished course of antibiotics is leaving the shelter before the storm has passed.	7	No	N/A	N/A	N/A	N/A	**Appropriate**
N6.3	An unfinished course of antibiotics is turning off the stove before the food is fully cooked.	7	No	N/A	N/A	N/A	N/A	**Appropriate**
N6.4	An unfinished course of antibiotics is a half-built bridge.	7	No	N/A	N/A	N/A	N/A	**Appropriate**
N6.5	An unfinished course of antibiotics is abandoning a train journey half-way.	5	Yes	N/A	4	Yes	N/A	Unsure

N6.6	An unfinished course of antibiotics is like quitting school before your final exams.	5	Yes	N/A	5	Yes	N/A	Unsure
N6.7	An unfinished course of antibiotics is like stopping your car in the middle of a busy motorway.	3.5	Yes	N/A	3	No	N/A	Inappropriate
N6.8	An unfinished course of antibiotics is like falling asleep at the cinema and missing the end of the film.	3.5	Yes	N/A	3	No	N/A	Inappropriate
N6.9	An unfinished course of antibiotics is like painting only half a wall.	5	No	N/A	4	No	N/A	Unsure
N6.10	An unfinished course of antibiotics is like investing money and never letting it mature.	3	No	N/A	N/A	N/A	N/A	Inappropriate
N6.11	An unfinished course of antibiotics is like turning off a video game without saving your progress.	4	Yes	N/A	3	No	N/A	Inappropriate
**Section 7: Following medical advice**								
N7.1	Ignoring medical advice is throwing darts in the dark.	5.5	Yes	N/A	5	No	N/A	Unsure
N7.2	Ignoring medical advice is walking around the house at night without turning on the lights.	5.5	Yes	N/A	5	No	N/A	Unsure
N7.3	Ignoring medical advice is fixing a car without a mechanic.	4.5	Yes	N/A	5	Yes	N/A	Unsure
N7.4	Ignoring medical advice is trying to build a rocket without an engineer.	6	No	N/A	7	No	N/A	**Appropriate**
N7.5	Ignoring medical advice is sailing into open waters without a captain or a compass.	7	No	N/A	N/A	N/A	N/A	**Appropriate**

N7.6	Ignoring medical advice is lighting a fire in your kitchen and assuming it won't spread.	3	Yes	N/A	3	No	N/A	Inappropriate
N7.7	Ignoring medical advice is building a house without a blueprint or an architect.	4.5	Yes	N/A	5	Yes	N/A	Unsure
N7.8	Ignoring medical advice is flying a plane without training.	7.5	No	N/A	N/A	N/A	N/A	**Appropriate**
N7.9	Ignoring medical advice is operating heavy machinery without reading the manual.	7	Yes	N/A	5	Yes	N/A	Unsure
N7.10	Ignoring medical advice is assembling flat-pack furniture without the instructions.	4	Yes	N/A	3	No	N/A	Inappropriate
N7.11	Ignoring medical advice is giving your friend a haircut using gardening shears.	3	No	N/A	N/A	N/A	N/A	Inappropriate
N7.12	Ignoring medical advice is using a hammer for every household repair.	3	No	N/A	N/A	N/A	N/A	Inappropriate
N7.13	You should follow medical advice just like you leave the bread to the baker.	3	No	N/A	N/A	N/A	N/A	Inappropriate
N7.14	Ignoring medical advice is asking your hairdresser to pull a tooth.	5	Yes	Ignoring medical advice is like trying to extract a tooth on your own—risky and best left to a professional.	6	No	N/A	Unsure
N7.15	Ignoring medical advice is handing over a map to someone who can't read it.	4	Yes	N/A	3	No	N/A	Inappropriate
N7.16	Ignoring medical advice is hiking without a guide.	4.5	Yes	Ignoring medical advice is climbing a dangerous mountain without a guide.	7	No	N/A	**Appropriate**
**Section 8: Antibiotic use for minor infections**								
N8.1	Using antibiotics for minor infections is like spraying your whole garden with	7	No	N/A	N/A	N/A	N/A	**Appropriate**

	weed killer to remove a couple of plants.							
N8.2	Using antibiotics for minor infections is calling the army to swat a fly.	6	Yes	N/A	6	Yes	N/A	Unsure
N8.3	Using antibiotics for minor infections is using the SWAT team for every little problem.	4.5	Yes	Using antibiotics for minor infections is using elite police teams to deal with minor wrongdoings.	5	Yes	N/A	Unsure
N8.4	Using antibiotics for minor infections is putting a plaster cast on a paper cut.	6	Yes	N/A	6	Yes	N/A	Unsure
N8.5	Using antibiotics for minor infections is using hurricane help in a light breeze.	4	Yes	N/A	3	No	N/A	Inappropriate
N8.6	Using antibiotics for minor infections is overwatering a healthy plant.	4.5	Yes	N/A	4	No	N/A	Unsure
N8.7	Using antibiotics for minor infections is charging a phone that's already full.	3.5	Yes	N/A	3	No	N/A	Inappropriate
N8.8	Using antibiotics for minor infections is visiting a surgeon for a scratch.	7	No	N/A	N/A	N/A	N/A	**Appropriate**
N8.9	Using antibiotics for minor infections is wearing winter coats in a summer drizzle.	5.5	Yes	N/A	5	Yes	N/A	Unsure
N8.10	Using antibiotics for minor infections is sending your best soldiers into battle every day.	3	Yes	N/A	2	No	N/A	Inappropriate
N8.11	Using antibiotics for minor infections is over sounding the emergency alarm.	4	Yes	N/A	4	Yes	N/A	Unsure
N8.12	Using antibiotics for minor infections is burning through emergency supplies.	5	Yes	N/A	5	Yes	N/A	Unsure
**Section 9: Infection prevention**								

N9.1	We can save antibiotics by avoiding infection, just we avoid drawing our swords by using shields.	3	No	N/A	N/A	N/A	N/A	Inappropriate
N9.2	We can save antibiotics by avoiding infection, just like we avoid endless mopping by fixing a leaky pipe.	7	No	N/A	N/A	N/A	N/A	**Appropriate**
N9.3	We can save antibiotics by avoiding infection, just like we can save firefighters by preventing outbreak of fire.	7	No	N/A	N/A	N/A	N/A	**Appropriate**
N9.4	We can save antibiotics by avoiding infection, just like we can save traffic police by maintaining good traffic management.	5	Yes	N/A	5	Yes	N/A	Unsure
N9.5	We can save antibiotics by avoiding infection, just like we can save ourselves trouble by locking our doors at night.	6	Yes	N/A	6	No	N/A	Unsure
N9.6	We can save antibiotics by avoiding infection, just like we avoid cavities through brushing our teeth.	7	No	N/A	N/A	N/A	N/A	**Appropriate**
N9.7	We can save antibiotics by avoiding infection, just like we protect ourselves from accidents by wearing seatbelts.	7.5	No	N/A	N/A	N/A	N/A	**Appropriate**
N9.8	We can save antibiotics by avoiding infection, just like we save our spare tyres through regular care maintenance.	6	No	N/A	6	No	N/A	Unsure

Supplementary Note 4: Metaphors consensually rated 'appropriate' by the e-Delphi expert panel

Note: The following metaphors were recommended by international AMR expert communicators for public health communications. They may be re-used, citing this publication, under CC BY 4.0 license.

Section 1: Diversity of microbial world

- The world of germs is a garden full of diverse insects - some helpful, a few dangerous.
- Communities of germs are like human neighbourhoods, made up of diverse residents including a few trouble-makers with the potential to cause chaos.
- Germs are as varied as animals, living in balance but fighting back to survive if threatened.

Section 2: Mechanisms of antibiotic resistance

- Weeds resist weed killer—bacteria resist antibiotics.
- Antibiotic-resistant bacteria are intruders wearing smart armour that upgrades itself when exposed to antibiotics.
- Antibiotic-resistant bacteria change the locks to effective treatment, making our antibiotic keys useless.
- Bacteria are like flowing water, finding creative ways around barriers.

Section 3: Antiviral efficacy

- Using antibiotics for viruses is using the wrong key to unlock a door.
- Using antibiotics for viruses is like putting diesel in a petrol car.
  Note: Depending on linguistic context, 'petrol' may need to be replaced with 'gas'.
- Using antibiotics for viruses is like entering the wrong password for your username.
- Using antibiotics for viruses is using the wrong charger for your phone.
- Using antibiotics for viruses is putting earphones over your eyes.
- Using antibiotics for viruses is changing the oil in your car when you have a flat tyre.
- Using antibiotics for viruses is using salt to sweeten your coffee.

Section 4: Body resistance

- Just like a bad apple spoils the whole bushel, a few resistant bacteria can affect the wider population—changing the balance and spreading resistance to others.
- Like a single missing roof tile can leave a room drenched, one resistant bacterium can compromise the protection antibiotics offer.

Section 5: Sharing of leftover antibiotics

- Sharing leftover antibiotics is using your house key for someone else's door.

Section 6: Self-directed premature termination of treatment course

Note: The metaphors in this section may need to be accompanied by information that antibiotics should be taken as directed. Shorter treatment durations can be both safe and appropriate, but require guidance from a qualified medical professional.

- An unfinished course of antibiotics is a fire half-out. Embers remain, ready to flare up again even stronger
- An unfinished course of antibiotics is a partially weeded garden with roots left behind that will grow back even more resilient.
- An unfinished course of antibiotics is a half-constructed house with no roof.
- An unfinished course of antibiotics is leaving the shelter before the storm has passed.
- An unfinished course of antibiotics is turning off the stove before the food is fully cooked.
- An unfinished course of antibiotics is a half-built bridge.

Section 7: Following medical advice

- Ignoring medical advice is baking without a recipe.
- Ignoring medical advice is trying to build a rocket without an engineer.
- Ignoring medical advice is sailing into open waters without a captain or a compass.
- Ignoring medical advice is flying a plane without training.
- Ignoring medical advice is climbing a dangerous mountain without a guide.

Section 8: Antibiotic use for minor infections

Note: The metaphors in this section may need to be accompanied by additional information including a definition of the term 'minor infection'—referring to infections that typically resolve without the need for antibiotic treatment.

- Using antibiotics for minor infections is using a sledgehammer to kill a fly.
- Using antibiotics for minor infections is using a hammer to crack a peanut.
- Using antibiotics for minor infections is using a fire extinguisher to blow out a candle.
- Using antibiotics for minor infections is like spraying your whole garden with weed killer to remove a couple of plants.
- Using antibiotics for minor infections is visiting a surgeon for a scratch.

Section 9: Infection prevention

- We can save our antibiotics by avoiding infection just like we avoid emergency services by maintaining our car.
- We can save antibiotics by avoiding infection, just like we avoid endless mopping by fixing a leaky pipe.
- We can save antibiotics by avoiding infection, just like we can save firefighters by preventing outbreak of fire.
- We can save antibiotics by avoiding infection, just like we avoid cavities through brushing our teeth.
- We can save antibiotics by avoiding infection, just like we protect ourselves from accidents by wearing seatbelts.

Supplementary Note 5: Research materials

Co-design

UK Co-design Workshops: Introductory presentation slides

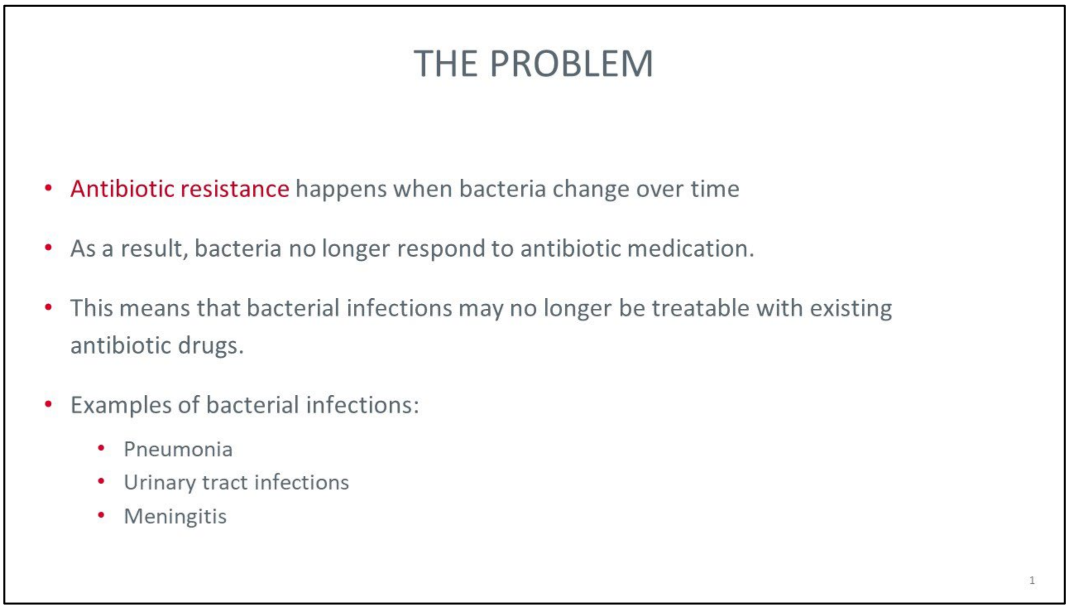

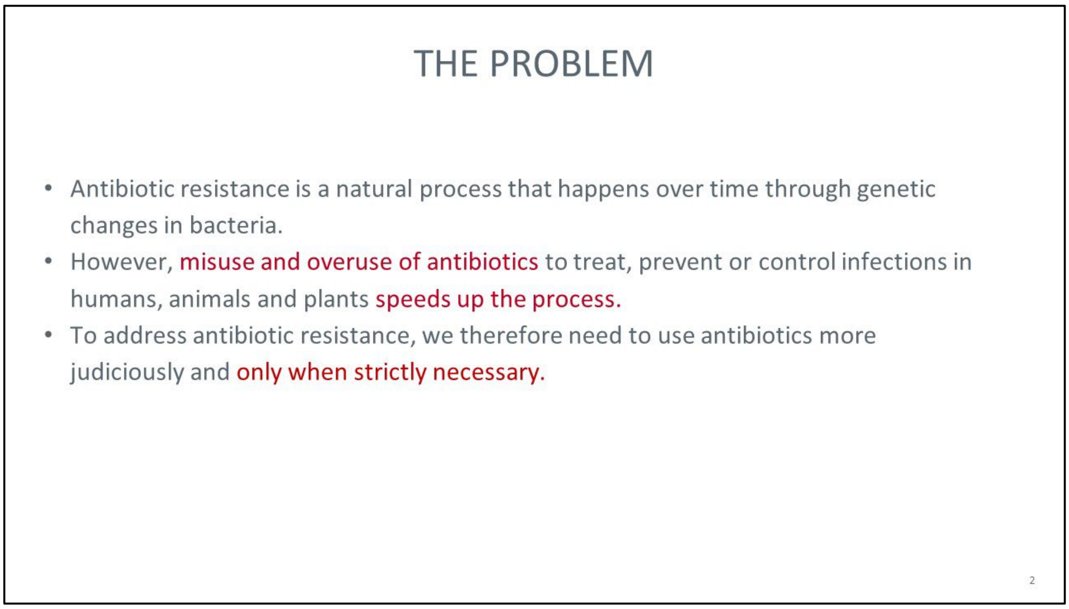

THE PROBLEM

- Previous research has shown that most people underestimate the risk of antibiotic resistance.
- Many people don't know about antibiotic resistance
- Even if people have heard of antibiotic resistance, many of them do not worry about it much in their day-to-day life.

3

PUBLIC MISUNDERSTANDINGS

- There are public misunderstandings related to antibiotics and antibiotic resistance.

1. Virus vs. Bacterial infection

- One misbelief is that antibiotics can be used to treat viral illnesses such as colds or flu.
- This is incorrect, because antibiotics are only effective for treating bacterial infections.
- This misunderstanding is problematic because people might end up expecting or demanding antibiotic medicines for illnesses such as colds when they aren't needed.

4

PUBLIC MISUNDERSTANDINGS

- There are public misunderstandings related to antibiotics and antibiotic resistance.

2. Transmission of resistance

- Another misbelief is that the body becomes resistant to antibiotics.
- This is incorrect because antibiotic resistance involves bacteria becoming resistant.
- This misunderstanding is problematic because it may mislead people to think that antibiotic resistance will not affect them personally if they—as an individual—do not use antibiotics.

5

RISK COMMUNICATION

Effective language and risk communication about antibiotic resistance should:

- be able to explain the abstract concept of antibiotic resistance
- draw attention to the health risks involved
- be simple and memorable so that people of different education levels can use the information
- address or correct common misunderstandings
- inspire people to take action, for example by sharing risk information with others or by adapting their behaviour.

6

METAPHORS

- One particular tool for health risk communication is the use of metaphors.
- Metaphors are illustrative language that draw comparisons between two different subject areas.
- They can highlight similarities across different domains
- This can help to improve people's understanding of abstract topics.

7

EXAMPLES OF METAPHORS

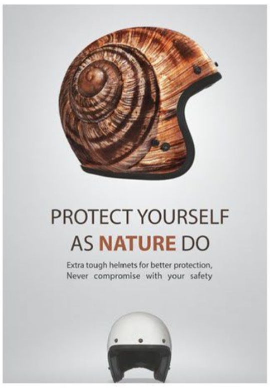

Metaphor:
Crash helmet = snail's shell

Meaning provided through metaphor:
Protection is a natural thing to do

Outcome:
People wear more helmets and get injured less.

8

EXAMPLES OF METAPHORS

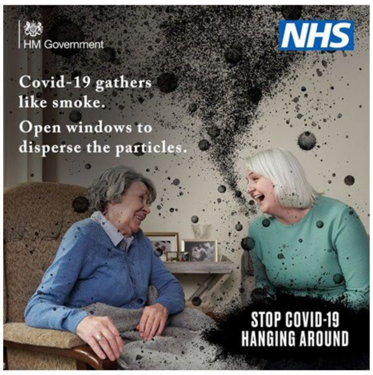

Metaphor:

COVID-19 = smoke

Meaning provided through metaphor:

COVID-19 particles are in the air around us

Outcome:

People open windows and get sick less often.

9

EXAMPLES OF METAPHORS

Cancerous tissue is like a garden that has become overgrown with weeds.

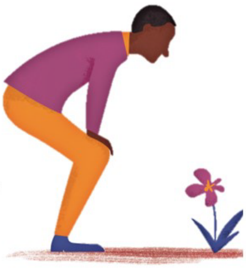

The weed-killer also damages the healthy plants, but you hope they will re-grow.

Metaphor:

Cancer tissue = garden weeds

Meaning provided through metaphor:

Aggressive treatment is necessary, but side-effects will be short-lived

Outcome:

People become accepting of necessary treatment and hopeful for the future.

10

METAPHORS

- The three previous metaphors are very different, but all have a few things in common:

1. They communicate information about a complex health topic: road safety, COVID-19 and cancer treatment
2. They do so by drawing a comparison between the complex health topic and a much simpler topic that most people are likely to be familiar with: snails, smoke and garden weeds.

11

INVITATION TO BRAINSTORM

- We would like to invite you to brainstorm some own ideas on terminology and metaphors in the context of antibiotics and antibiotic resistance.
- If possible, these metaphors should help to communicate one of the following messages
    1. Antibiotic resistance is a severe health threat to all people.
    2. Antibiotics can only treat bacterial infections. They don't treat viral infections (such as the flu) and only cause unnecessary side effects if used for viral infections.
    3. Bacteria become resistant to antibiotics, not people. Resistant bacteria spread from one person to another, and this means that anybody can get ill from a drug-resistant infection—even if they've never taken antibiotics in their life.

12

CREATING A METAPHOR

Problem - Solution - Call to Action

Visualised in a smart, imaginative way

13

CREATING A METAPHOR

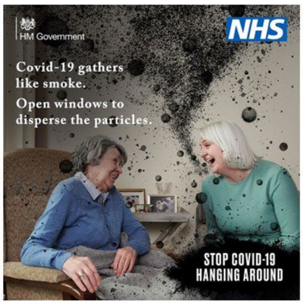

Problem - Solution - Call to Action

Virus - Air flow - Open the window

Words - Stop Covid-19 hanging around
Visualisation - smoke

14

CREATING A METAPHOR

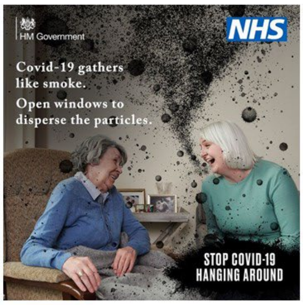

Metaphor dimensions to consider:

Emotionality: How might metaphors create emotional reactions?

Aptness: Do metaphors communicate something relevant about the topic?

Systemicness: Do they provide a coherent framework to shape our understanding and subsequent choices?

15

CREATING A METAPHOR

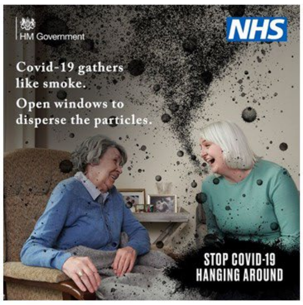

Metaphor dimensions to consider:

Emotionality: How might metaphors create emotional reactions?

Smoke is associated with a threat. Dark smoke looks menacing.

Aptness: Do metaphors communicate something relevant about the topic?

Systemicness: Do they provide a coherent framework to shape our understanding and subsequent choices?

16

CREATING A METAPHOR

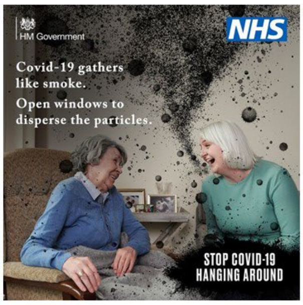

Metaphor dimensions to consider:

Emotionality: How might metaphors create emotional reactions?

Aptness: Do metaphors communicate something relevant about the topic?

COVID-19 lingers in the air – just like smoke.

Systemicness: Do they provide a coherent framework to shape our understanding and subsequent choices?

17

CREATING A METAPHOR

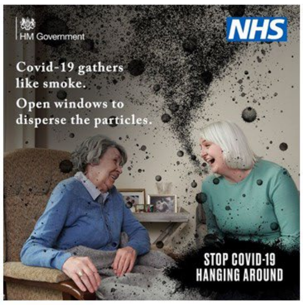

Metaphor dimensions to consider:

Emotionality: How might metaphors create emotional reactions?

Aptness: Do metaphors communicate something relevant about the topic?

Systemicness: Do they provide a coherent framework to shape our understanding and subsequent choices?
COVID-19 can be dispersed by opening windows – just like smoke.

18

CREATIVE EXERCISES

What you need:

- Pen/pencil
- Paper / notepad
- Online/offline doc to make notes

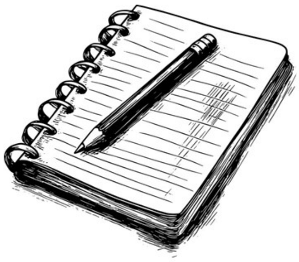

19

South African Co-design Workshop: Introductory presentation slides

THE PROBLEM

- Antibiotic resistance happens when bacteria change over time
- As a result, bacteria no longer respond to antibiotic medication.
- This means that bacterial infections may no longer be treatable with existing antibiotic drugs.
- Examples of bacterial infections:
  - Pneumonia
  - Urinary tract infections
  - Meningitis

1

THE PROBLEM

- Antibiotic resistance is a natural process that happens over time through genetic changes in bacteria.
- However, misuse and overuse of antibiotics to treat, prevent or control infections in humans, animals and plants speeds up the process.
- To address antibiotic resistance, we therefore need to use antibiotics more judiciously and only when strictly necessary.

2

DISCUSSION

- What kind of misconceptions in your context do people have about AMR?

3

METAPHORS 'IZIFANISO'

- One particular tool for health risk communication is the use of metaphors.

  'Intsebenziso yezifaniso sesinye isixhobo kunxibelelwano lweengozi zempilo.'

- Metaphors are illustrative language that draw comparisons between two different subject areas.

  'Izifaniso yindlela ekusetyenziswa ngayo ulwimi lwembonakaliso ngokuthelekisa izifundo ezimbini ezahlukileyo.'

- They can highlight similarities across different domains

  'Zikwazi ukuveza ukufana kwezinto ezikumasebe ohlukileyo'

- This can help to improve people's understanding of abstract topics.

  'Oku kunganceda ukuphucula indlela abantu abaqonda ngayo izihloko ezintsokothileyo'

5

EXAMPLES OF METAPHORS 'UMZEKELO WEZIFANISO'

Ukunwenwa kwesifo sephepha kunjengomlilo okhawulezayo endaweni enengca eyomileyo.

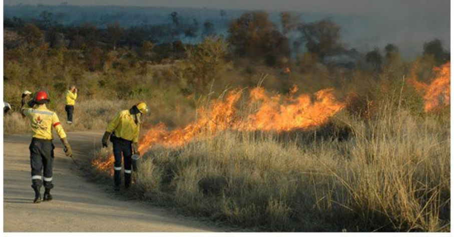

Metaphor:

Umlilo = Isifo sephepha

Meaning provided through metaphor:

Ukunwenwa kwesifo sephepha kunjengomlilo wedobo

Outcome:

Abantu balumkele ubungozi bokusuleleka

6

EXAMPLES OF METAPHORS

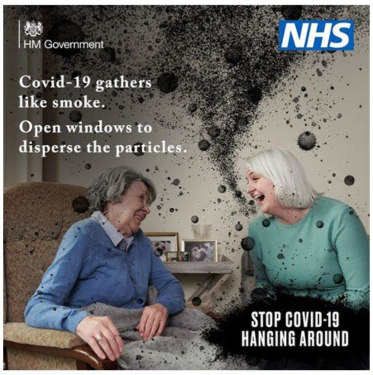

Metaphor:
COVID-19 = smoke

Meaning provided through metaphor:
COVID-19 particles are in the air around us

Outcome:
People open windows and get sick less often.

7

EXAMPLES OF METAPHORS

Cancerous tissue is like a garden that has become overgrown with weeds.

The weed-killer also damages the healthy plants, but you hope they will re-grow.

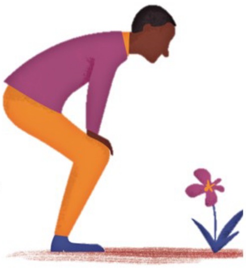

Metaphor:
Cancer tissue = garden weeds

Meaning provided through metaphor:
Aggressive treatment is necessary, but side-effects will be short-lived

Outcome:
People become accepting of necessary treatment and hopeful for the future.

8

METAPHORS

- The three previous metaphors are very different, but all have a few things in common:

1. They communicate information about a complex health topic: TB, COVID-19 and cancer treatment
2. They do so by drawing a comparison between the complex health topic and a much simpler topic that most people are likely to be familiar with: fire, smoke and garden weeds.

9

CREATING A METAPHOR
'UKWAKHA ISIFANISO'

Problem - Solution - Call to Action

Ingxaki – Isisombululo – ubizelo esenzweni

Visualised in a smart, imaginative way

Imbonakaliso ngendlela ekrelekrele nenoyilo

10

CREATING A METAPHOR

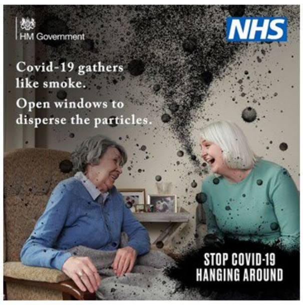

Problem - Solution - Call to Action

Virus - Air flow - Open the window

Words - Stop Covid-19 hanging around
Visualisation - smoke

11

CREATING A METAPHOR

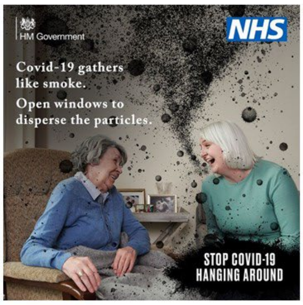

Metaphor dimensions to consider:

Emotionality/Imvakalelo: How might metaphors create emotional reactions?

Aptness/Ukufaneleka: Do metaphors communicate something relevant about the topic?

Systemicness/Ucwangco: Do they provide a coherent framework to shape our understanding and subsequent choices?

12

CREATING A METAPHOR

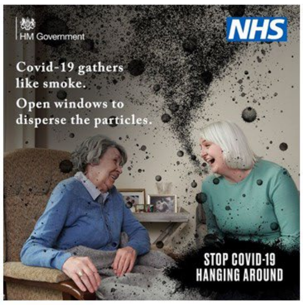

Metaphor dimensions to consider:

Emotionality/Imvakalelo: How might metaphors create emotional reactions?
Smoke is associated with a threat. Dark smoke looks menacing.

Aptness/Ukufaneleka: Do metaphors communicate something relevant about the topic?

Systemicness/Ugcwangco: Do they provide a coherent framework to shape our understanding and subsequent choices?

13

CREATING A METAPHOR

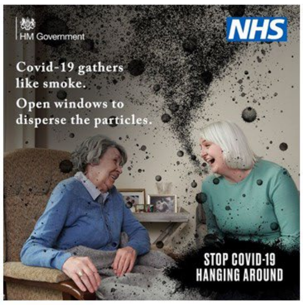

Metaphor dimensions to consider:

Emotionality/Imvakalelo: How might metaphors create emotional reactions?

Aptness/Ukufaneleka: Do metaphors communicate something relevant about the topic?
COVID-19 lingers in the air – just like smoke.

Systemicness/Ugcwangco: Do they provide a coherent framework to shape our understanding and subsequent choices?

14

CREATING A METAPHOR

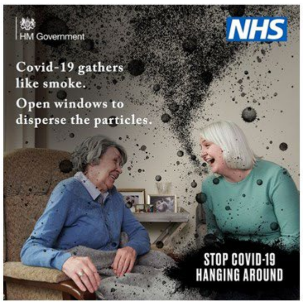

Metaphor dimensions to consider:

Emotionality/Imvakalelo: How might metaphors create emotional reactions?

Aptness/Ukufaneleka: Do metaphors communicate something relevant about the topic?

Systemicness/Ucwangco: Do they provide a coherent framework to shape our understanding and subsequent choices?

COVID-19 can be dispersed by opening windows – just like smoke.

15

CREATIVE EXERCISES

What you need:

- Pen/pencil
- Paper / notepad

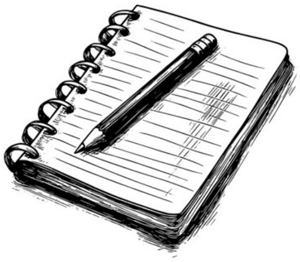

16

e-Delphi

Background information materials for e-Delphi study

Thank you for agreeing to participate in this study! Below, we provide some background to the study, list the aims and clarify the procedure. Please read the information carefully and get in touch with the research team in case of any questions.

Background to this study

A major challenge in addressing antimicrobial resistance (AMR) is the **general lack of public awareness**, which stems from ineffective communication. Research indicates that many people may not fully understand the risks associated with AMR or hold misconceptions, such as believing that antibiotics are effective against viral illnesses or that the body becomes resistant to antibiotics, rather than the bacteria (Shahpawee et al., 2020; Singh-Phulgenda et al., 2023). Ignorance and misbeliefs can give rise to inappropriate behaviours, which may exacerbate the problem. These include demands for antibiotics when they are not warranted, sharing antibiotics, and the failure to follow treatment recommendations (e.g., to complete a full treatment course), (McCullough et al., 2015).

Effective risk communication about AMR is essential to increase awareness, dispel myths and optimise behaviours. Public-facing communication about AMR may comprise traditional news media, social media and health messaging campaigns. Research into effective AMR communication is an emerging field, but initial investigations suggest that **existing messages fail to achieve the desired impact**.

Media reporting about AMR has been criticised for its superficial, attention-grabbing narratives about superbugs and dirty hospitals (Catalán-Matamoros et al., 2019), which often lacks recommendations regarding individual risk reduction measures (Bohlin & Höst, 2014). Compared to related health risks such as sepsis, AMR messages rarely incorporate patient stories to enhance personal relevance (Fitzpatrick et al., 2019; Rush et al., 2019). The frequent use of hyperbolic, apocalyptic imagery may be counterproductive, leading to de-sensitisation and apathy (Nerlich, 2009; Servitje, 2019), and overly simplistic war narratives (e.g., calling for a fight against evil bacteria) in newspapers and social media may misrepresent the nuanced reality of human-bacteria relationships (Bouchoucha et al., 2019; Krockow, 2025; Peters et al., 2019).

Several reviews have aimed to evaluate the effectiveness of public education campaigns. Although some campaigns showed measurable success (Gilham et al., 2024), many campaigns failed to achieve sustained behaviour change (Fletcher-Miles et al., 2020). The most successful campaigns used disease-specific messages targeted to selected socio-demographic groups, simple materials that aided recall, multiple media channels and interactive elements such as pledges or school activities.

Some research has used experimental approaches and hypothetical vignettes to test the effectiveness of specific AMR message elements for reducing antibiotic expectations amongst the general public. Simply explaining that antibiotics are ineffective against viral illnesses had an effect (Thorpe et al., 2021). Additionally, information designed to promote empathy (Santana et al., 2023) or fear (Roope et al., 2020; Sirota & Juanchich, 2024) could reduce patient demand for antibacterials, whereas labelling AMR as a 'silent' pandemic may dampen risk perceptions and willingness to take action (Sirota, 2024). An in-depth, mixed-methods evaluation of AMR message framing (Wellcome Trust, 2019) highlighted many problematic aspects of current AMR narratives,

including inconsistent use of language and a lack of personal relevance for present-day decision making. Follow-up experiments reinforced this, showing that inconsistent, abstract terms like 'AMR' and 'antimicrobial resistance' were ineffective in public communications (Grailey, 2025; Krockow et al., 2023)

Our own recent qualitative work with patients and doctors in the UK (Krockow et al., under review) identified that AMR messages fall short, because people find them personally irrelevant and are not motivated to engage in more depth. They are often also unable to engage given that information is perceived as very scientific, abstract and difficult to understand. Participants also perceived existing AMR messages as lacking in 'punch' and overall impact.

Taken together, the literature suggests that we need impactful new AMR messages that motivate people to engage and help them understand the abstract concept of AMR.

An **underexplored tool for engaging communication is metaphor**. By drawing comparisons between abstract and more familiar topics, it has the potential to shape the way people think and act.

Common metaphor examples are "time is *money*", "the exam was a *piece of cake*" and "life is a *journey*". Metaphors are powerful tools that can influence people's attitudes, opinions and behaviours. For example, research has shown that comparing crime to a *virus infecting a city* as opposed to a *wild beast preying on a city* led people to show more support for educational solutions as opposed to harsh punishment of offenders (Thibodeau & Boroditsky, 2011)

Metaphors have been successfully used to communicate about other health risks. For example, recent UK health campaigns compared COVID-19 to smoke to help people understand the importance of opening windows.

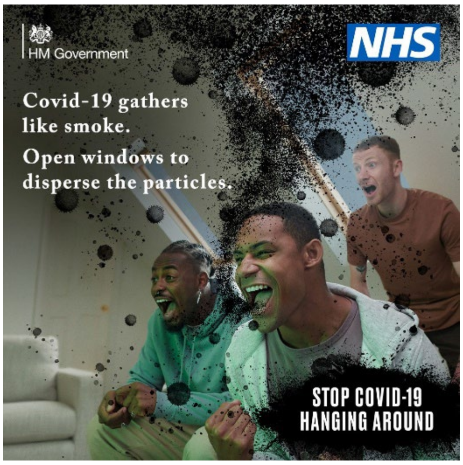

Metaphors are frequently used in AMR communications, with examples including: "war against evil superbugs", "silent pandemic", and "post-antibiotic apocalypse", but our recent work (Krockow et al., 2025) highlights that most metaphors create a misleading image of a simple battle between good and evil. They often fail to reflect AMR as an ongoing, evolving challenge or portray all bacteria as dangerous bugs, even though many of them are essential to our survival. Almost all existing metaphors are highly emotive (e.g., aiming to evoke a sense of urgency and doom). They might help to raise awareness about the risk of AMR but run the risk of desensitising audiences. They also typically fail to communicate specific knowledge or measures that can be translated into positive behaviour change.

When communicating with the general public, we need metaphors that are:

- **Apt** (i.e., able to capture and communicate a relevant aspect of AMR such as the fact that antibiotic overuse can exacerbate resistance or that resistance can spread from one individual to another)
- **Universal** (i.e., familiar and easy to relate to, no matter the person's upbringing, education or culture)
- **Persuasive** (i.e., able to challenge misunderstandings and inspire action such as better adherence to treatment advice)

Generating new metaphor ideas

Prior to this Delphi study, we engaged patients, doctors and members of the public in the UK and South Africa in interactive co-design workshops to brainstorm new metaphor ideas. We conducted 7 smaller workshops in the UK with a total of 29 participants and one larger workshop in South Africa (conducted in English and isiXhosa) with 22 participants. The comparatively small sample size and convenience sampling means that participants were not representative of the general population in the two countries, but we achieved some diversity in terms of age, gender, culture and education levels. Overall, the workshops produced a long list of novel metaphor ideas to improve communications about AMR. These metaphors offer a starting point for the current Delphi study.

Metaphor ideas can categorised based on the AMR problem aspect or misbelief that they address. For example, some metaphors communicate the general concept of AMR, while others address specific misbeliefs like the misconception that antibiotics can be used to treat viruses.

In the next step, we would like to invite you—as international AMR communication expert—to review and rate the initial metaphor ideas and contribute your own ideas. **By the end of this study, we hope to identify a shortlist of 3-6 metaphors with high potential for use in public health risk communication about AMR internationally.**

Below is an overview of the planned procedure. You will complete up to four study rounds over the next 2-3 months. The exact number will depend on how long it takes to reach an expert consensus about which AMR metaphors are likely to be most effective. We currently anticipate the following structure, but small changes may be possible:

- **Round 1 (20-30 minutes to complete)** – You will review and rate an initial list of metaphors for AMR communication and offer your own additional ideas.
- **Round 2 (15-20 minutes to complete)** – You will re-rate items after receiving feedback on the group's average ratings. You will also rate the metaphors that were newly suggested by yourself and other Delphi panel members.
- **Following rounds (15-20 minutes to complete)** – You will re-rate items after receiving feedback on the group's average ratings. Low-rated options will be removed to refine the shortlist.

Instructions for e-Delphi Round 1

Prior to this study, we engaged members of the public, patients and doctors in the UK and South Africa to brainstorm ideas of metaphors as a baseline for this study (see information above). You will be presented with these metaphor ideas shortly, and asked to rate their appropriateness. You will also be asked to think of additional metaphors. Some guiding principles and examples are provided below.

Appropriateness of Metaphors

When communicating with the general public, we need metaphors that are **appropriate**. Specifically, we are looking for metaphors that are:

- **Apt** (i.e., able to capture and communicate a relevant aspect of AMR such as the fact that antibiotic overuse can exacerbate resistance or that resistance can spread from one individual to another)
- **Universal** (i.e., familiar and easy to relate to, no matter the person's upbringing, education or culture)
- **Persuasive** (i.e., able to challenge misunderstandings and inspire action such as better adherence to treatment advice)

Below are some examples of how we might rate appropriateness of specific metaphors.

Example 1: *Covid-19* gathers like *smoke*.
The metaphorical comparison is Covid-19 = smoke.

- The metaphor is **apt** because it captures a relevant aspect of COVID-19; i.e., the virus hangs around in the air like smoke.
- The metaphor is **universal**, because almost everybody will know what smoke is.
- The metaphor is **persuasive**, because it inspires the action to open windows.

Considering these three aspects, one might draw the conclusion that the metaphor "*Covid-19* gathers like *smoke"* is **highly appropriate**. On a rating scale from 1-9, one might rate the metaphor as follows:

								■
1<br>Highly<br>Inappropriate	2	3	4	5	6	7	8	9<br>Highly<br>Appropriate

Example 2: *AMR* is a *silent pandemic*.
The metaphorical comparison is AMR = silent pandemic.

- The metaphor is **somewhat apt**. While many people aren't aware of AMR, many others are aware because they have already suffered its consequences. By calling it "silent", people are likely to underestimate the risk associated with AMR.
- The metaphor is **highly universal**. Following COVID-19, most people will be familiar with the concept of a pandemic.
- The metaphor is **not persuasive**, because it doesn't address any specific knowledge gaps or misunderstandings, not does it inspire a specific action.

Considering these three aspects and weighing up their relative importance, one might draw the conclusion that whilst universal, the metaphor *"AMR* is a *silent pandemic"* is unlikely to b persuasive, subsequently giving it an overall rating of '**somewhat inappropriate'**. On a rating scale from 1-9, one might rate the metaphor as follows:

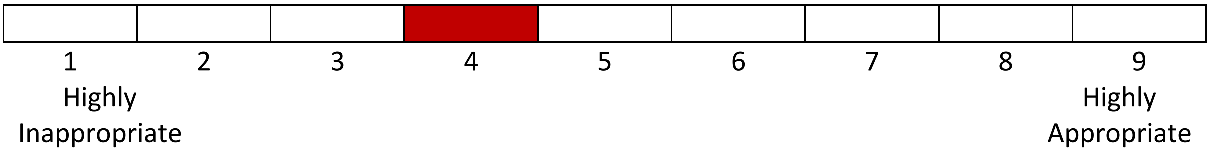

Example 3: "Antibiotics are *silver bullets*"

The metaphorical comparison is Antibiotics = silver bullets.

- The metaphor is **inapt**. While it captures the way in which especially broad-spectrum antibiotics can provide a quick and effective treatment for severe infection or sepsis, it may mislead people to think that antibiotics can cure all ailments. It also downplays the risk of AMR.
- The metaphor is **somewhat universal**. The concept of a bullet is universal but not everybody is likely to understand "silver" in combination with "bullet". Overall, the understanding may depend on context.
- The metaphor is **not persuasive** for positive behaviour change, because highlighting the power of antibiotics could be counterproductive. It could encourage problematic behaviours, such as demands for antibiotics when they aren't necessary or inappropriate self-medication.

Considering these three aspects, one might draw the conclusion that the metaphor "Antibiotics are *silver bullets"* is **inappropriate**. On a rating scale from 1-9, one might rate the metaphor as follows:

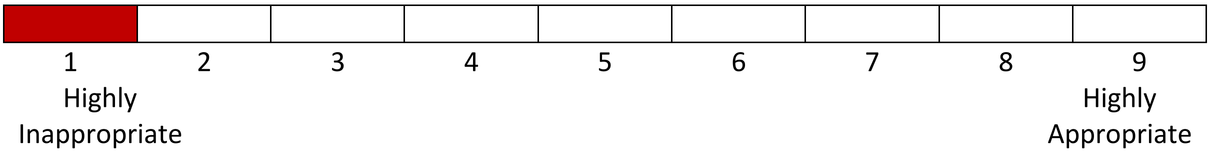

Instructions for e-Delphi Round 2

We will now ask you to re-rate some of the metaphors from Round 1. We have only included those metaphors, where there was uncertainty regarding appropriateness. We have also included a few metaphors previously rated as appropriate, where the wording was changed following comments from Round 1.

Please note that following feedback from the first round, all metaphors have been **shortened significantly for succinctness and punch**, while an effort was made to retain the core ideas.

Additionally, we have tried— where possible and appropriate—to **opt for more direct metaphorical comparisons** as opposed to the use of analogies or similes. **A metaphor** says one thing *is* another to

create imagery, **a simile** compares two things using *like* or *as*, and **an analogy** explains a relationship or concept by showing how two things are similar in structure or function. Following participant feedback from Round 1, we tried to use metaphors where possible to create more vivid imagery.

For the purpose of re-rating, we will show you the groups' median rating as well as your own previous ratings. In some cases, we will show you a summary of relevant qualitative comments from the group for your consideration. We would like you to re-rate all items, reflecting on the groups' score, comments and your previous opinions.

To resolve uncertainty and shorten the metaphor list for re-rating in the next round, we suggest that you choose decisive rating scores (either very high ratings or very low ratings), where possible.

General group recommendations from the previous round:

Across your ratings, please also consider the following general group feedback around effective metaphors:

- We need to identify metaphors that work across a **wide range of different contexts** and can be easily translated.
- We need to use **simple language** so it can be understood by people of lower education levels.
- We should aim for **vivid and direct comparisons** that make an impact.
- **Short metaphors** might be easier to remember than long ones.

Instructions for e-Delphi Round 3

Thank you very much for completing the second round of the Delphi study, especially given the very large item number!

Aims of Round 3

The aim of this Delphi study is to develop a **curated list of metaphors** endorsed by international experts, to be shared via an electronic 'metaphor menu'. This resource will serve as a messaging toolkit for global AMR communicators—including international organisations, charities, governments, healthcare institutions, and individuals. Selected metaphors may also be evaluated in a follow-up experiment for their potential to influence attitudes and behaviours.

Ideally, by the end of Round 3, we want to **arrive at a clear consensus** about which metaphors are appropriate for communication about AMR and with aren't. Thus, ideally, we want to:

- reduce disagreement between experts
- reduce the number of items rated 'unsure' (between 4-6 on the appropriateness scale).

Structure of Round 3

The third Delphi round will consist of 67 items. These items were retained for another round of ratings because their scores had one or more of the following characteristics:

- There was disagreement among participants about the appropriateness of an item, indicated by a large spread of scores or by a bi-modal distribution (where some participants liked the item a lot, while others disliked it).
- Participants felt unsure about an item's appropriateness, indicated by an average (median) score that fell within the range of 4-6 on the appropriateness scale.

To achieve this, the questionnaire will be structured as follows:

- We will present all remaining items within the original section structure.
- Each section will be pre-empted by some general, summarised feedback from participants and additional considerations and instructions shared by the research team.
- Additionally, for each item, we will present you with the groups' median rating as well as your own previous rating. In some cases, we will show you a summary of item-specific comments or inform you in case an item has been re-worded following previous participant feedback.
- Again, we will ask for section-specific comments or any additional explanations that you have alongside your quantitative ratings.

Rating guidance for Round 3

When re-rating the items, please consider all of the information presented, reflecting on the groups' score, comments and your previous opinions.

To resolve uncertainty and avoid the necessity for a fourth round of ratings, we suggest that you choose decisive rating scores (either very high ratings or very low ratings), where possible.

Please consider keep in mind the previous guidance on how to rate appropriateness of metaphors. Specifically, we are looking for metaphors that are:

- **Apt** (i.e., able to capture and communicate a relevant aspect of AMR such as the fact that antibiotic overuse can exacerbate resistance or that resistance can spread from one individual to another)
- **Universal** (i.e., familiar and easy to relate to, no matter the person's upbringing, education or culture)
- **Persuasive** (i.e., able to challenge misunderstandings and inspire action such as better adherence to treatment advice)

Example of e-Delphi item rating on Clinvivo Platform

Section one: The microbial world is diverse

These metaphors tackle the misbelief that all germs are the same. They lay the groundwork to understanding that antibiotics are not a solution to all infectious diseases, but only work against specific pathogens.

*

1a. The microbial world is like a box of chocolates. Some chocolates are nice, some nasty - just like the different types of microbes.

Highly inappropriate — Highly appropriate

1	2	3	4	5	6	7	8	9

*

1b. The microbial world is like a range of mushrooms - some tasty, others dangerous. Similarly, some microbes are beneficial, while others are dangerous.

Highly inappropriate — Highly appropriate

1	2	3	4	5	6	7	8	9

*

1c. The microbial word is like an invisible rain forest with lots of diverse flora and fauna – invisible to the naked eye and with beautiful diversity.

Highly inappropriate — Highly appropriate

1	2	3	4	5	6	7	8	9
